# Synthesis of Novel Benzothiazole–Profen Hybrid Amides as Potential NSAID Candidates

**DOI:** 10.3390/molecules30010107

**Published:** 2024-12-30

**Authors:** Iliyan Ivanov, Stanimir Manolov, Dimitar Bojilov, Yordan Stremski, Gabriel Marc, Stela Statkova-Abeghe, Smaranda Oniga, Ovidiu Oniga, Paraskev Nedialkov

**Affiliations:** 1 Department of Organic Chemistry, Faculty of Chemistry, University of Plovdiv, 24 “Tsar Assen” Str., 4000 Plovdiv, Bulgaria; manolov@uni-plovdiv.bg (S.M.); bozhilov@uni-plovdiv.net (D.B.); stremski@uni-plovdiv.bg (Y.S.); stab@uni-plovdiv.bg (S.S.-A.); 2 Department of Organic Chemistry, Faculty of Pharmacy, “Iuliu Hațieganu” University of Medicine and Pharmacy, 41 Victor Babeș Street, RO-400010 Cluj-Napoca, Romania; marc.gabriel@umfcluj.ro; 3 Department of Pharmaceutical Chemistry, Faculty of Pharmacy, “Iuliu Hațieganu” University of Medicine and Pharmacy, 41 Victor Babeș Street, RO-400010 Cluj-Napoca, Romania; smaranda.oniga@umfcluj.ro; 4 Department of Therapeutic Chemistry, “Iuliu Hațieganu” University of Medicine and Pharmacy, 12 Ion Creangă Street, RO-400010 Cluj-Napoca, Romania; ooniga@umfcluj.ro; 5 Department of Pharmacognosy, Faculty of Pharmacy, Medical University of Sofia, 2 Dunav Street, 1000 Sofia, Bulgaria; pnedialkov@pharmac.mu-sofia.bg

**Keywords:** benzothiazole, profens, hybrid molecules, amides, in vitro biological activity, lipophilicity, molecular docking, molecular dynamics

## Abstract

Herein, we report the synthesis of a series of new compounds by combining 2-aminobenzothiazole with various profens. The compounds were characterized using techniques such as ^1^H- and ^13^C-NMR, FT-IR spectrometry, and high-resolution mass spectrometry (HRMS), with detailed HRMS analysis conducted for each molecule. Their biological activities were tested in vitro, revealing significant anti-inflammatory and antioxidant effects, comparable to those of standard reference compounds. Lipophilicity was experimentally determined through partition coefficient (R_M_) measurements. To understand their binding affinity, molecular docking studies were perfsormed to analyze interactions with human serum albumin (HSA). The stability of these predicted complexes was further evaluated through molecular dynamics simulations. The results highlight the compounds’ promising biological activity and strong affinity for HSA. The new hybrid molecule between 2-ABT and ketoprofen **3b** demonstrates significant promise based on the experimental data and is further supported by in silico calculations. Compound **3b** exhibits the best hydrogen peroxide scavenging activity among the tested compounds, with an IC_50_ of 60.24 μg/mL. Furthermore, **3b** also displays superior anti-inflammatory activity, with an IC_50_ of 54.64 μg/mL, making it more effective than the standard ibuprofen (76.05 μg/mL).

## 1. Introduction

Inflammation and oxidative stress are central to the pathology of numerous chronic conditions, including arthritis, cardiovascular diseases, and neurodegenerative disorders. Despite the widespread use of non-steroidal anti-inflammatory drugs (NSAIDs), current treatments often come with limitations, such as gastrointestinal and cardiovascular side effects, highlighting the pressing need for effective and safer alternatives [1]. In this context, hybrid molecules that combine the anti-inflammatory properties of classic NSAIDs with other pharmacophores that possess antioxidant activity offer a promising therapeutic path [2].

Benzothiazole derivatives have emerged as versatile scaffolds in medicinal chemistry due to their broad spectrum of biological activities, including anti-inflammatory and antioxidant properties [3,4]. Notably, compounds such as 2-aminobenzothiazole (2-ABT) are widely used as a precursor for the synthesis of a variety of hybrid compounds with proven pharmacotherapeutic properties such as anti-inflammatory, anticancer, antioxidant, antibacterial, etc. [4,5,6].

**Figure 1:**
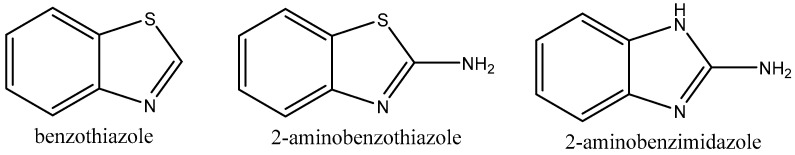
Structural formulas of benzothiazole, 2-aminobenzothiazole, and 2-aminobenzimidazole.

**Figure 2:**
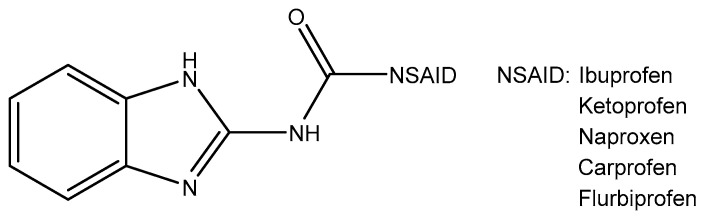
New 2-ABZ-NSAID conjugates as potential drug candidates.

Benzothiazole (BT) and its derivatives (Figure 1) are privileged organic scaffolds with a wide range of biomedical activities, particularly in the development of therapeutic agents [7]. In addition, during the last decade, the functionalization of BT scaffold has modulated a significant number of potential chemotherapeutic agents, highlighting its versatility and importance in medicinal chemistry [8,9,10,11,12,13]. Notably, compounds such as 2-ABT (Figure 1) and 2-aminobenzimidazole (2-ABZ) (Figure 1) are widely used as precursors for the synthesis of a variety of hybrid compounds with proven pharmaco-therapeutic referrals [14,15,16,17,18,19].

2-ABT is highly versatile in multicomponent reactions (MCRs) because it can act in different roles, such as a nucleophile, an electrophile, or a Michael donor, depending on the reaction conditions and the other components involved. Recently, it has been widely used in making various heterocycles, including isoxazoles, pyrazoles, imidazoles, pyridines, indoles, and fused heterocycles. These applications have led to new efficient methods for quickly building complex molecules [20].

The amino group in 2-ABT makes it weakly basic, meaning it can accept a proton to result in a positively charged ammonium ion. The respective compound has aromatic properties because its π-electrons are spread across both the benzene and thiazole rings, which affects how it reacts in chemical reactions. 2-ABT can undergo electrophilic aromatic substitution, where a hydrogen atom on the aromatic ring is replaced by another group [21,22]. The amino group can also bind to metal ions, forming stable complexes, which changes the compound’s properties and reactivity [23].

Profens are a category of non-steroidal anti-inflammatory drugs (NSAIDs) that are widely used for their pain-relieving, anti-inflammatory, and fever-reducing properties. They have several disadvantages and potential risks, particularly when used improperly or for prolonged periods [24].

Chemists use 2-ABT as a starting material to create complex organic molecules. Its structure, with an amino group and a BT ring, makes it versatile for making compounds with various properties, including those used in medicine, agriculture, and materials science [20]. This compound is an important building block for the development of heterocyclic systems and fused ring structures. It can interact with other compounds in reactions either by attracting or contributing electron pairs, depending on the context, enabling its involvement in processes such as condensation, cyclization, and MCRs. These reactions make it easy to quickly create complex molecules from simple materials in one step [20].

Hybrid molecules containing two pharmacophores are designed to integrate the therapeutic benefits of distinct pharmacophore entities into a single molecule, enhancing their pharmacological profile [25,26,27]. This approach enables simultaneous modulation of multiple biological targets, making it a promising strategy for complex diseases where multiple pathways contribute to pathophysiology, such as inflammation, oxidative stress, etc. [28]. In this regard, Banzal and Silakari reported the synthesis of new 2-ABZ-NSAID conjugates (Figure 2) with anti-inflammatory, immunomodulatory, and antioxidant activities [29].

The synthesis of amides is important for drug discovery, where it is estimated that amide preparation is essential [30]. In this regard, approximately a quarter of all marketed drugs and two-thirds of all drug candidates contain at least one amide functional group [31]. Taking into account how amides interact with biological targets is also a key aspect from a pharmaceutical point of view [32].

Based on these findings and considering the structural and electronic similarities that allow BT to act as an isostere of benzimidazole in medicinal chemistry, in this article, we propose the synthesis of some new BT–profen hybrid molecules as potential NSAID candidates. By combining the properties of BTs, known for their antioxidant and anti-inflammatory activities, with those of profens, which are widely used NSAIDs, the hybrid molecules can offer synergistic effects, potentially improving efficacy while reducing side effects.

The linker designed to connect the two pharmacophore fragments is an amide group while considering the possible interactions between the amide and biological target: the ability of amide groups to form multiple interactions (e.g., hydrogen bonds and van der Waals forces) often stabilizes the binding of a molecule to its target.

## 2. Results and Discussion

### 2.1. Synthesis

**Scheme 1:**
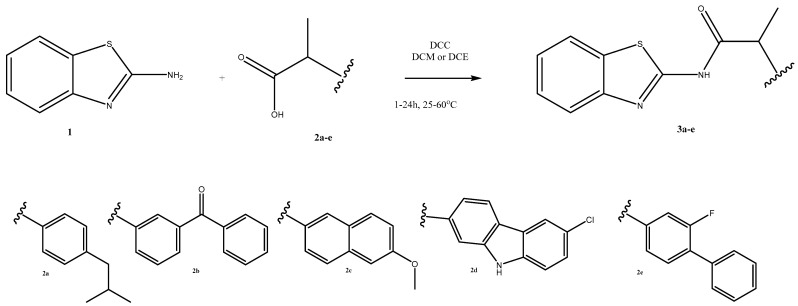
Synthetic approach for the synthesis of benzothiazole–profen hybrids **3a**–**e**.

This study explores the synthesis of five amides using a straightforward and accessible procedure involving *N*,*N*′-dicyclohexylcarbodiimide (DCC)—mediated coupling between 2-ABT **1** and various profens **2a**–**e** as carboxylic acid precursors (Scheme 1). In the literature, this method is well-established for the synthesis of diverse organic compounds [33]. The reaction conditions were optimized for the use of DCC as a coupling agent to produce the compounds **3a**–**e**, as illustrated in Scheme 1.

Dichloromethane (DCM) is a suitable solvent for carrying out the reactions, but 1,2-dichloroethane (DCE) was found to be the optimal solvent for the reaction with carprofen **2d** due to its low solubility in DCM. The yield of the products depended on the solubility of the profen in the used solvent. The resulting products were obtained in good yields, from 71% to 92%, verified to be pure and structurally characterized by ^1^H, ^13^C-NMR, IR, and MS spectra. Compound **3e** was reported previously by us (see Section 3.2).

NMR analysis confirmed the full structure of the obtained molecules **3a**–**e**. The observed signals of the ^1^H-NMR spectra correspond with all protons in the expected structures. For all the compounds, the proton from the NH group is shifted at the range of δ = 12.16–12.65 ppm, and the signals for -CH (quartet) and -CH_3_ (doublet) from the profens can be seen at the range of δ = 4.04–4.23 and δ = 1.49–1.52 ppm, respectively. The signals in the ^13^C-NMR spectra also matched the number of carbon atoms in the analyzed molecules. The IR spectra of all the compounds showed a single band at about 3300 cm^−1^ for the -NH- group in secondary amines. Finally, the high-resolution mass spectrum of each compound showed the base peak at the *m/z* value, corresponding to the theoretical accurate mass of its parent ion [M+H]^+^ ion at the positive mode using electrospray ionization (ESI). Original spectra are available in the Supplementary Materials section.

### 2.2. In Vitro Biological Assessment

#### 2.2.1. Hydrogen Peroxide Scavenging Activity

Copper (Cu^2+^) and iron (Fe^2+^) ions play critical roles as cofactors in numerous enzymes and physiological processes within the human body. However, in their free state, these ions can be harmful. Cu^2+^ ions, for instance, accelerate the oxidation of ascorbic acid, leading to the generation of reactive oxygen species (ROS) such as superoxide radicals (O_2_^•−^) and hydrogen peroxide (H_2_O_2_). Furthermore, through the Fenton reaction, Cu^2+^ and Fe^2+^ ions react with H_2_O_2_ to produce highly reactive hydroxyl radicals (^•^OH) [34]. Oxidative stress is a key factor in the development of various health issues, including cancer, cardiovascular disease, atherosclerosis, and Alzheimer’s disease [35].

In addition to oxidative stress mechanisms, the inflammatory process also contributes to the production and amplification of ROS. The generation of superoxide anion radicals is a primary cause of inflammation, and it is closely associated with the formation of other reactive oxygen species, such as hydrogen peroxide (H_2_O_2_) [36].

**Figure 3:**
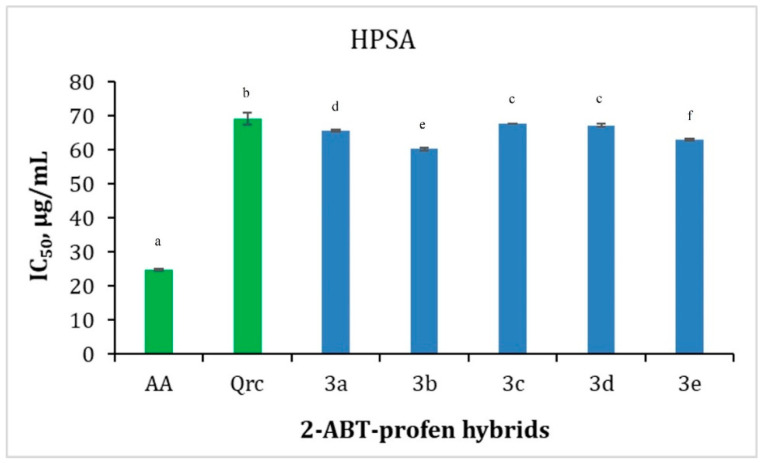
Results of the HPSA of 2-ABT derivatives **3a**–**e**. Values are presented as IC_50_, µg/mL. Ascorbic acid (AA) and quercetin (Qrc) were used as standards. Different letters for the same method indicate a significant difference at the *p* < 0.05 level by Duncan’s test. Duncan’s test compares the average values of the groups and classifies them by significance levels. Group average values that do not differ significantly are united into homogeneous subgroups.

Therefore, in the present work, we investigated the ability of 2-ABT derivatives to inhibit the damaging effects of H_2_O_2_. The natural antioxidants ascorbic acid and quercetin were used as comparative standards. Figure 3 illustrates the data from the hydrogen peroxide scavenging activity (HPSA) assay, with the IC_50_ values of the compounds provided in Table S1 of the Supplementary Materials section. The IC_50_ values of the synthesized compounds ranged between 60.24 and 67.71 µg/mL. Compared to ascorbic acid, the 2-ABT derivatives exhibited lower antioxidant activity. Statistical evaluation of the antioxidant activity of 2-ABT derivatives was performed using Duncan’s range test. Duncan’s range test allows for the assessment of differences in antioxidant activity while also assessing how the activity varies depending on the structure of the compounds. Compound **3b**, incorporating a ketoprofen fragment, exhibited a lower IC_50_ value than the standard quercetin (Figure 3 and Table S1), demonstrating higher antioxidant activity relative to quercetin and the other derivatives.

#### 2.2.2. Hydroxyl Radical Scavenging Activity (HRSA)

The hydroxyl radical (^•^OH) is the most powerful oxidizing agent, generated as part of reactive oxygen species (ROS). It forms through one-electron reductions of molecular oxygen (O_2_) during cellular metabolism and serves as the main driver of cytotoxicity in aerobic organisms, including humans [37].

Highly reactive ^•^OH radicals cause protein carbonylation, DNA damage, and cell membrane disruption, and are responsible for the oxidation of essential fatty acids, a process known as lipid peroxidation.

**Figure 4:**
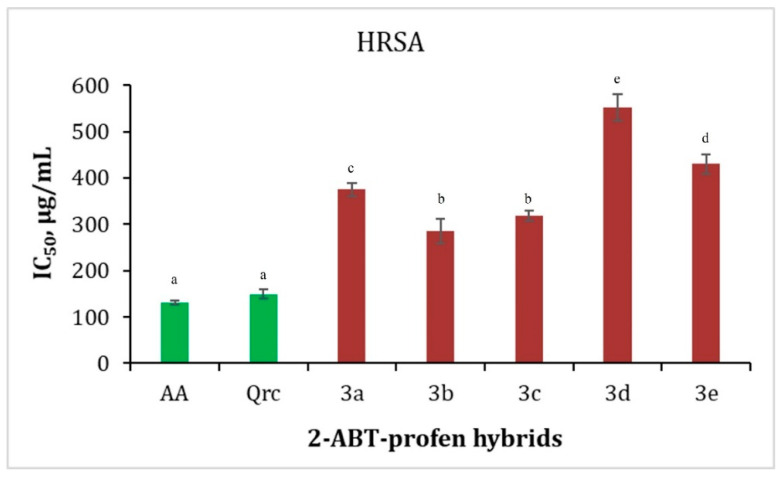
Results of the HRSA of 2-ABT derivatives **3a**–**e**. Values are presented as IC_50_, µg/mL. Ascorbic acid (AA) and quercetin (Qrc) were used as standards. Different letters for the same method indicate a significant difference at the *p* < 0.05 level by Duncan’s test. Duncan’s test compares the average values of the groups and classifies them by significance levels. Group average values that do not differ significantly are united into homogeneous subgroups.

Compared to ascorbic acid (24.84 µg/mL) and quercetin (69.25 µg/mL), the synthesized 2-ABT-profen hybrids exhibited lower in vitro antioxidant activity (Figure 4 and Table S1). Figure 4 highlights a significant variation in antioxidant activity among the tested compounds. The results were analyzed using Duncan’s test, a statistical method to determine significant differences between groups. According to the test, different letters above the columns of each compound indicate statistically significant differences. Hybrid molecules **3b** and **3c**, which contain ketoprofen and naproxen residues, respectively, showed significantly lower IC_50_ values compared to the other derivatives, indicating higher antioxidant activity against hydroxyl radicals (Figure 4 and Table S1). This notable antioxidant activity suggests potential applications in combating oxidative stress.

#### 2.2.3. Inhibition of Albumin Denaturation (IAD)

Inflammation is the process by which living tissues respond to stimuli caused by inflammatory factors such as physical damage, heat, microbial infections, and harsh chemical irritants. The response of the cells to inflammation will lead to certain pathological manifestations, characterized by redness, heat, swelling, and pain, and even impaired physiological functions. Numerous disorders, including arthritis, stroke, and cancer, include inflammation as a pathogenic factor. Protein denaturation is closely linked to the initiation of the inflammatory response, which results in a variety of inflammatory disorders, including arthritis [38]. According to Opie [39], tissue injury during life might be caused by the denaturation of the protein constituents of cells or of intercellular substances. Hence, the ability of a substance to inhibit the denaturation of protein signifies apparent potential for anti-inflammatory activity.

**Figure 5:**
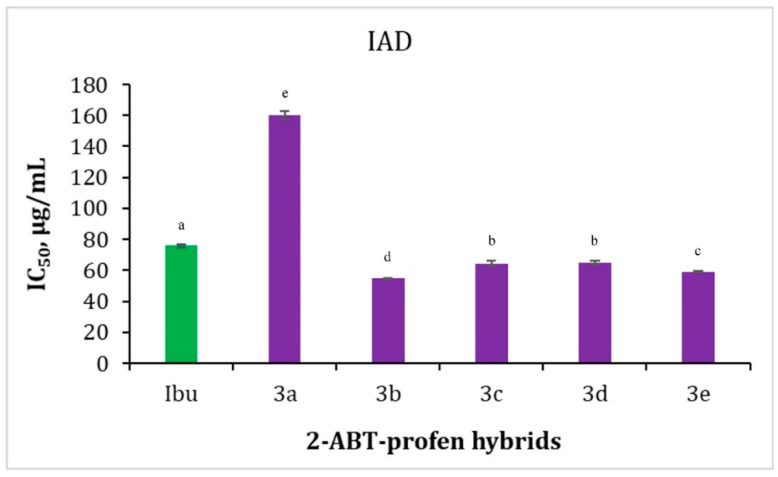
Results of the IAD of 2-ABT derivatives **3a**–**e**. Values are presented as IC_50_, µg/mL. Ibuprofen (Ibu) was used as a standard. Different letters for the same method indicate a significant difference at the *p* < 0.05 level by Duncan’s test. Duncan’s test compares the average values of the groups and classifies them by significance levels. Group average values that do not differ significantly are united into homogeneous subgroups.

Figure 5 presents the inhibition percentages of the synthesized 2-ABT-profen hybrids, with results expressed as IC_50_ values. Ibuprofen, a widely used anti-inflammatory drug, served as a reference for evaluating the activity of the newly synthesized 2-ABT derivatives. The IC_50_ value of ibuprofen, calculated as IAD, is 76.05 µg/mL (Figure 5 and Table S1). The IC_50_ values for compounds **3a**–**e** range from 54.64 to 159.94 µg/mL (Figure 5 and Table S1). Notably, compounds **3b**–**e** exhibit significantly higher activity compared to **3a** and ibuprofen. Furthermore, a clear structure–activity relationship is observed: an increase in the number of aromatic rings and the aromatic nature of the profen fragments correlates with enhanced activity. These findings highlight the potential of certain 2-ABT derivatives as anti-inflammatory agents, underscoring their importance for future medical and pharmaceutical applications.

#### 2.2.4. Lipophilicity

Lipophilicity is a crucial physicochemical property in the development of new drugs, significantly influencing their absorption, distribution, metabolism, excretion, and toxicity (ADMET) profiles. In drug discovery, maintaining optimal lipophilicity is essential for ensuring that drug candidates possess desirable pharmacokinetic and pharmacodynamic properties.

Recent studies indicate that there has been a trend toward increasing lipophilicity in drug candidates over the years, which can lead to several adverse effects, such as poor aqueous solubility and increased metabolic turnover. For instance, compounds with log *p* values exceeding Lipinski’s rule-of-five criteria often exhibit undesirable characteristics like high plasma protein binding and potential toxicity [40,41]. This change raises concerns about finding the right balance between making the drug strong enough to work effectively and keeping its physical and chemical properties suitable. To tackle these challenges, researchers are focusing on using lipophilic efficiency measures in drug design. These measures help balance a drug’s fat-loving properties (lipophilicity) while keeping it effective. Tools like reversed-phase liquid chromatography are now commonly used to quickly and accurately measure lipophilicity, making it easier to make better decisions early in drug development [42].

Lipophilicity can be evaluated through calculations or experimental methods. In our study, we measured the lipophilicity of the synthesized 2-ABT derivatives using a practical method: reversed-phase thin-layer chromatography, as described by Hadjipavlou-Litina [43]. The results are shown in Table S1. The lipophilicity increased in the following order based on the experimentally obtained RM values: **3a** (1.629) < **3d** (1.755) < **3e** (1.736) < **3b** (1.941) < **3c** (2.02). Compound **3c** is the most lipophilic experimentally, followed by **3b**, while **3a** is the least lipophilic. This trend suggests variability in the lipophilic character of the obtained compounds, likely due to differences in their functional groups or molecular structures. Compounds with higher *R_M_* values exhibit better interaction with lipid membranes [44].

#### 2.2.5. MS Spectral Characterization

The newly synthesized hybrids **3a**–**d** contain 2-ABT in their structure. The essential difference is that the structure of the new 2-ABT derivatives contains the profen fragment. Mass spectral analysis shows that the amide bond is energetically weaker, and under ESI-MS conditions, we established two fragmentation pathways of the molecular ion—cleavage of the C(O)-C(C) bond (**pathway 1**) and the amide bond (N-C(O)) (**pathway 2**) (Scheme 1).

**Scheme 2:**
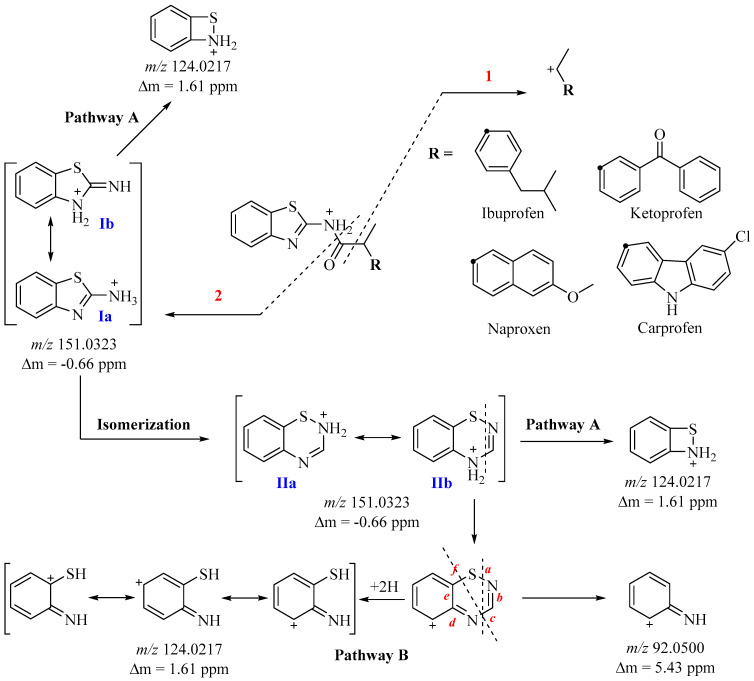
General fragmentation pathway of the newly synthesized 2-ABT derivatives **3a**–**e**.

Pathway 1 involves the cleavage of the C(O)-C(C) bond and leads to the formation of a resonance-stable profen cation—*m/z* 161 for ibuprofen, *m/z* 209 for ketoprofen, *m/z* 185 for naproxen, and *m/z* 228 for carprofen (Scheme 2 and Figures S15, S18, S21 and S24). The mechanism of pathway 2 is more complex. Initially, the amide bond is cleaved and leads to the formation of a resonance-stable benzo[*d*]thiazol-2-aminium cation (*m/z* 151) (canonical structure **Ia** and **Ib**). The same ion, under ESI-MS/MS conditions, can probably isomerize to the 2H-benzo[*e*][1,2,4]thiadiazin-2-ium resonance-stable cation (canonical structure **IIa** and **IIb**) (Scheme 2), which potentially explains its high intensity.

From the canonical structures **Ib** and **IIb**, through the loss of 27 Da (HCN), a biradical cation is initially obtained, which, after cyclization, leads to the formation of a stable 7-thia-8-azabiciclo[4.2.0]octa-1,3,5-trien-8-ium cation *m/z* 124 (**pathway A**) (Scheme 2). The same ion (*m/z* 124) is probably obtained by the isomerization of canonical structure **IIb** and subsequent retrocyclization with the cleavage of the *σ*-bonds a and c and acceptance of two protons (+2H) to obtain a monocyclic resonance-stable cation (**pathway B**). Furthermore, retrocyclization with the cleavage of the bonds c and f leads to the formation of an ion with *m/z* 92 (Scheme 2).

#### 2.2.6. Molecular Docking

The results of the molecular docking of the R and S enantiomers of compounds **3a**–**e** to the albumin binding sites, as obtained using AutoDock Vina, are summarized in Table 1, whereas the results obtained with AutoDock are shown in Table 2, presented as the top binding conformation found in the most populated cluster of conformations for each compound, to ensure the reproducibility of the results.

By comparing the binding values for each site (Sudlow I, Sudlow II, site III, and cleft), it is possible to identify the relative preference of compounds for specific binding sites as Sudlow II, where higher binding affinities were generally found across it, and site III, which was in second place. For the two respective sites, the studied compounds consistently showed the strongest binding affinities in most cases. For the cleft site, an intermediate affinity of compounds **3a**–**e** was identified, while for the Sudlow I site, they exhibited the lowest affinity. The respective binding pattern was identified in the results from both AutoDock (Table 1) and AutoDock Vina (Table 2).

Across compounds, the S isomers consistently showed slightly stronger binding affinities to all studied binding sites than the R isomers, suggesting that stereochemistry may play a role in the interaction strength of the compounds with albumin.

Based on the analysis of data, compound **3b** appears to have the most promising binding among the current series of compounds, with consistently strong binding affinities across all sites, especially its S isomer. Compound **3b** exhibited the best binding for Sudlow’s site I and site III according to the analysis of the results of the molecular docking provided by AutoDock Vina. However, the results of AutoDock did not confirm the previous findings, with the binding affinity of all compounds being close to each other in a specific site.

On the other hand, compound **3a** might be the least effective for HSA binding, considering its relatively weaker binding affinity compared to the other compounds after analyzing the AutoDock Vina results and for Sudlow’s site II and the cleft site after analyzing the AutoDock results.

Since the data obtained from the molecular docking results were not conclusive, it was necessary to confirm the stability of the complexes through a molecular dynamics study, described in the next section.

**Figure 6:**
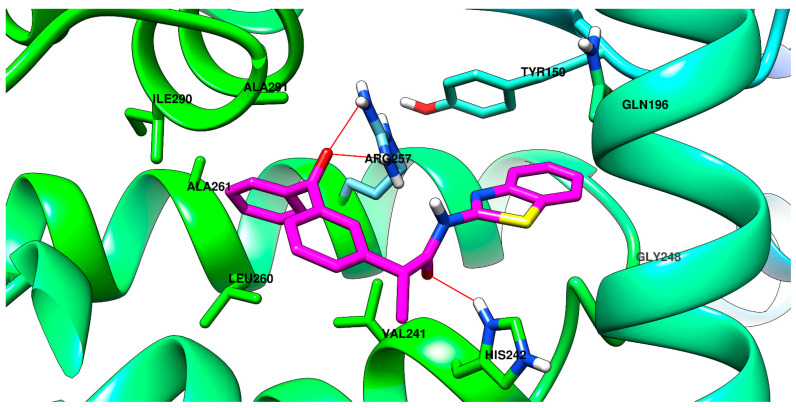
The top-ranked conformation of compound **3b[R]** (carbon atoms depicted in magenta) bound in Sudlow’s site I of albumin. Among the current series of compounds, **3b[R]** showed the strongest binding to this site. Two ion–dipole interactions (depicted in red) are predicted to appear between the amide oxygen atom and Hys242 sidechain and between the ketone oxygen atom and Arg257.

**Figure 7:**
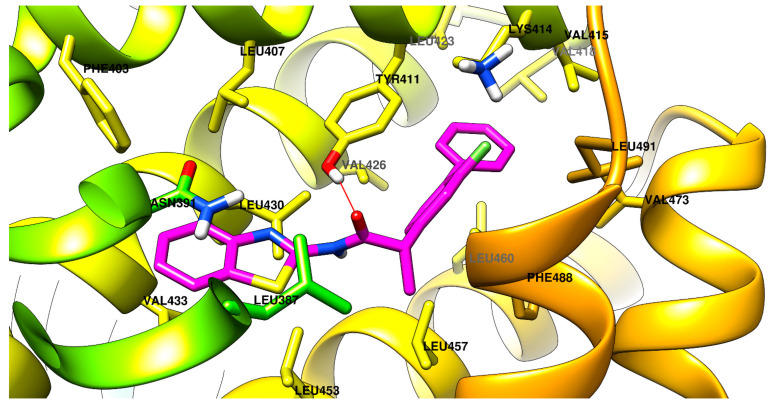
The top-ranked conformation of compound **3e[S]** (carbon atoms depicted in magenta) bound in Sudlow’s site II of albumin. Among the current series of compounds, **3e[S]** showed the strongest binding to this site. A hydrogen bond (depicted in red) is predicted to appear between the amide oxygen atom as an acceptor and the Tyr411 sidechain as a donor.

**Figure 8:**
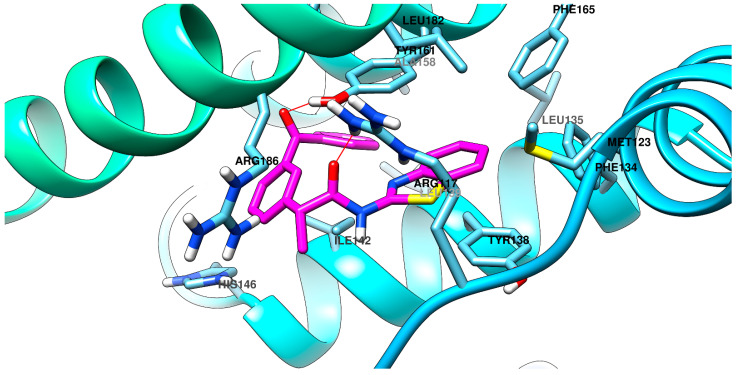
The top-ranked conformation of compound **3b[R]** (carbon atoms depicted in magenta) bound in site III of albumin. Among the current series of compounds, **3b[R]** showed the strongest binding to this site. An ion–dipole interaction (depicted in red) is predicted to appear between the amide oxygen atom and Arg117 sidechain, and a hydrogen bond (depicted in red) is predicted to appear between the ketone oxygen atom as an acceptor and Tyr161 as a donor.

**Figure 9:**
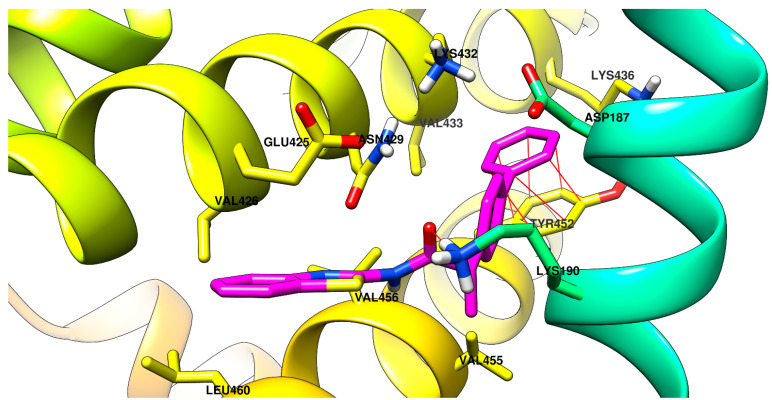
The top-ranked conformation of compound **3e[S]** (carbon atoms depicted in magenta) bound in the cleft site of albumin. Among the current series of compounds, **3e[S]** showed the strongest binding to this site. An ion–dipole interaction (depicted in red) is predicted to appear between the amide oxygen atom and Lys190 sidechain and a π–π stacking (depicted in red) is predicted to appear between the terminal phenyl of **3e[S]** and Tyr452.

For all top binding conformations of the compounds, at least one strong binding interaction was observed between the ligand and albumin—a hydrogen bond or a π–cation interaction. The respective bonds are depicted in Figure 6, Figure 7, Figure 8 and Figure 9 by red lines. Most of the other interactions of ligands with albumin are predicted to occur with the hydrophobic sidechains of amino acids found in the binding site.

For each binding site studied, the top binding predicted the pose of the compound, which exhibited the best binding affinity to the respective site, as depicted in Figure 6, Figure 7, Figure 8 and Figure 9, respectively.

#### 2.2.7. Molecular Dynamics

The stability of the albumin complexes with the top binding conformation in each of the four studied binding sites was evaluated in the molecular dynamics study. The stability of the protein–ligand simulated systems in the molecular dynamics study was expressed by calculating the root mean square deviation (RMSD) of the backbone of the protein, the average root mean square deviation (RMSD) of the heavy atoms of ligands, the radius of gyration (RG) of the protein and the hydrogen bonds between the ligand and the protein.

The results from the molecular dynamics analysis are summarized as follows: Table 3 provides the RMSD values for ligand heavy atoms, Table 4 displays the RMSD values for the protein backbone in the apo state and in complex with ligands, Table 5 shows the radius of gyration (RG) for the protein in the apo state and in complex with ligands, and Table 6 provides the average number of hydrogen bonds formed between albumin and the ligands.

The RMSD of the albumin backbone is lower in the cases where a ligand was bound to any of the four binding sites compared to that of the apo state, suggesting that ligand binding at the four sites contributes to a stabilizing effect on albumin. The movement of the bound ligand was the smallest for **3e[S]** in the Sudlow II site (0.15 nm) and **3b[R]** in the Sudlow I site (0.21 nm), indicating a tight and stable binding for the respective ligands. Increased movement was identified for compound **3e[S]** in the cleft site (0.27 nm) and an instability was identified for compound **3b[R]** docked in site III of HSA after 60 ns of simulation, but with a correction in the last ns of the simulation. For the respective compound, the higher RMSD of the heavy atoms indicates potential instability of the complex, but a longer simulation would be necessary to confirm this. The RG of the protein was increased for albumin when **3b[R]** was docked in site III (2.80 nm), which is in agreement with the previous observation (Figures S25–S28). For the rest of the studied complexes, the RG of the protein was lower or at least equal to the apo state. Hydrogen bonding was found to be involved in ligand complexation in the cleft site more than the others, which can be attributed to the particularities of the binding site more so than to the compound’s structure.

## 3. Materials and Methods

### 3.1. General

All reagents and chemicals were purchased from commercial sources, namely, Sigma-Aldrich and Merck (Darmstadt, Germany). ^1^H-, ^13^C-NMR spectra were measured using a Bruker Avance II+ 600 spectrometer (Bruker, Billerica, MA, USA) at BAS-IOCCP, Sofia, and chemical shifts (δ, ppm) were downfield from TMS. For adequate assignment of peaks and structure determination, the spectra were measured at 80 °C in DMSO-*d_6_*, as indicated below. IR spectra were measured on a VERTEX 70 FT-IR spectrometer (Bruker Optics, Ettlingen, Germany). The reactions were monitored by thin-layer chromatography on precoated 0.2 mm Merck silica gel 60 plates. Silica gel, high-purity grade, 60, 100–200 mesh was used for column chromatography purification.

A Millipore purifier (Millipore, Burlington, MA, USA) was used to obtain water for HPLC. Potassium dihydrogen phosphate, dipotassium hydrogen phosphate, phosphoric acid, potassium chloride, quercetin, ibuprofen, sodium chloride, hydrogen peroxide, sodium salicylate, ferrous sulfate, Tween 80, and DMSO were purchased from Sigma-Aldrich, Taufkirchen, Germany. Human albumin 20%—BB, 200 g/L was ordered from BB-NCIPD Ltd., Sofia, Bulgaria.

### 3.2. Synthesis

*N*,*N*′-Dicyclohexylcarbodiimide (DCC—1 mmol, 0.206 g) was added to a solution of the corresponding profen (1 mmol) in 10 mL dichloromethane (DCM). For the reaction with carprofen, 15 mL 1,2-dichloroethane (DCE) was used instead because of the poor solubility of the profen in DCM. The reaction mixture was stirred at room temperature for 30 min. After the addition of 2-aminobenzothiazole (1 mmol, 0.150 g), the reaction mixture was stirred under different conditions: for 4 h at 25 °C in the case of **3a**; for 5 h at 25 °C for **3b** and **3c**; and for 24 h, starting at 25 °C and increasing to 60 °C, for **3d**. The completion of the reactions was monitored by TLC. The formation of white crystalline dicyclohexylurea (DHU) was observed and then separated by filtration over a sintered glass filter, and the solvent was removed at reduced pressure. The ethyl acetate (10 mL) was added to the residue and filtrated again to remove the remaining DHU. The filtrate was washed with a diluted hydrochloric acid (10%), a saturated solution of Na_2_CO_3_ (3%), and brine. The combined organic layers were dried over anhydrous Na_2_SO_4_, and the solvent was removed under reduced pressure. The compounds were purified by filtration through short column chromatography on silica gel.

**3a (*N*-(benzo[*d*]thiazol-2-yl)-2-(4-isobutylphenyl)propanamide)**

Pale yellow oil, yield of 78% (0.2636 g), isolated by column chromatography on silica gel with mixtures of petroleum/diethyl ether (3:1, increasing polarity to 2:1). ^1^H-NMR (600 MHz, DMSO-*d*_6_, 80 °C, δ ppm) 12.16 (brs, 1H, NH, amide), 7.92 (d, *J* = 7.9 Hz, 1H, CH, Ar, benzothiazole), 7.71 (d, *J* = 8.1 Hz, 1H, CH, Ar, benzothiazole), 7.41 (t, *J* = 7.0 Hz, 1H, CH, Ar, benzothiazole), 7.21–7.36 (m, 3H, 2xCH, Ar, ibuprofen, CH, Ar, benzothiazole), 7.07–7.16 (m, 2H, 2xCH, Ar, ibuprofen), 4.04 (q, *J* = 7.0 Hz, 1H, *CH, ibuprofen), 2.43 (d, *J* = 7.0 Hz, 2H, CH_2_, ibuprofen), 1.71–1.95 (m, 1H, CH(CH_3_)_2_, ibuprofen), 1.49 (d, *J* = 7.0 Hz, 3H, CH_3_, ibuprofen), 0.86 (d, *J* = 6.6 Hz, 6H, CH(CH_3_)_2_, ibuprofen). ^13^C-NMR (151 MHz, DMSO-*d*_6_, 80 °C, δ ppm) 173.9 (-C=O, amide), 158.5 (CH, Ar), 149.1 (CH, Ar), 140.4 (CH, Ar), 138.5 (CH, Ar), 132.1 (CH, Ar), 129.5 (CH, Ar), 129.3 (CH, Ar), 127.6 (CH, Ar), 127.5 (CH, Ar), 126.4 (CH, Ar), 123.9 (CH, Ar), 121.9 (CH, Ar), 120.9 (CH, Ar), 45.3 (CH_2_, ibuprofen), 44.8 (*CH, ibuprofen), 29.8 (CH(CH_3_)_2_, ibuprofen), 22.6 (CH(CH_3_)_2_, ibuprofen), 18.7 (CH_3_, ibuprofen). IR (KBr) ν_max_, cm^−1^: 3327 ν(N-H), 2928, 2851 ν(C_sp_^2^-H), 1701 ν(C=O), 1626 δ(N-H), 1573 ν(C=C, Ph), 1537 δ(N-H), ν(C-N), 1483 ν(C=C, Ph), 1436 δ_as_(CH_3_), δ(N-CH_2_), 1244, 1229 ν(C-N), 892, 779 γ(C_sp_^2^-H), 641 ν(C-S-C). HRMS electrospray ionization (ESI) *m/z* calcd for [M+H]^+^ C_20_H_23_N_2_OS^+^ = 339.1526, found 339.1521 (mass error ∆m = −1.47 ppm).

**3b (*N*-(benzo[d]thiazol-2-yl)-2-(3-benzoylphenyl)propanamide)**

White crystals (m.p. 113–114 °C), yield of 76% (0.2936 g), isolated by column chromatography on silica gel with a mixture of petroleum/diethyl ether 1:1. ^1^H-NMR (600 MHz, DMSO-*d*_6_, 80 °C, δ ppm) δ 12.32 (brs, 1H, NH, amide), 7.93 (d, *J* = 8.2 Hz, 1H, CH, Ar, benzothiazole), 7.83 (s, 1H, CH, Ar, benzothiazole), 7.70–7.76 (m, 4H, CH, Ar), 7.60–7.68 (m, 2H, CH, Ar), 7.50–7.56 (m, 3H, CH, Ar), 7.42 (t, *J* = 7.0 Hz, 1H, CH, Ar), 7.29 (t, *J* = 7.0 Hz, 1H, CH, Ar), 4.19 (q, *J* = 7.0 Hz, 1H, *CH, ketoprofen), 1.55 (d, *J* = 7.0 Hz, 3H, CH_3_, ketoprofen). ^13^C-NMR (151 MHz, DMSO-*d*_6_, 80 °C, δ ppm) 196.0 (C=O, ketoprofen), 141.7 (C=O, amide), 137.9 (CH, Ar), 137.7 (CH, Ar), 132.9 (CH, Ar), 132.0 (CH, Ar), 129.9 (CH, Ar), 129.2 (CH, Ar), 129.1 (CH, Ar), 129.0 (CH, Ar), 128.8 (CH, Ar), 126.5 (CH, Ar), 124.01 (CH, Ar), 122.0 (CH, Ar), 121.0 (CH, Ar), 45.5 (*CH, ketoprofen), 18.6 (CH_3_, ketoprofen). IR (KBr) ν_max_, cm^−1^: 3296 ν(N-H), 3060, 2926 ν(C_sp_^2^-H), 1700 ν(C=O), 1651 δ(N-H), 1596 ν(C=C, Ph), 1541 δ(N-H), ν(C-N), 1441 ν(C=C, Ph), 1381 δ_as_(CH_3_), δ(N-CH_2_), 1288, 1265 ν(C-N), 821, 755 γ(C_sp_^2^-H), 642 ν(C-S-C). HRMS electrospray ionization (ESI) *m/z* calcd for [M+H]^+^ C_23_H_19_N_2_O_2_S^+^ = 387.1162, found 387.1158 (mass error ∆m = −1.03 ppm).

**3c (*N*-(benzo[*d*]thiazol-2-yl)-2-(6-methoxynaphthalen-2-yl)propanamide)**

White crystals (m.p. 65–66 °C), yield of 74% (0.2689 g), isolated by column chromatography on silica gel with mixtures of petroleum/diethyl ether (2:1, increasing polarity to 1:1). ^1^H-NMR (600 MHz, DMSO-*d*_6_, 80 °C, δ ppm) 12.26 (s, 1H, NH, amide), 7.92 (d, *J* = 7.8 Hz, 1H, CH, Ar, benzothiazole), 7.77–7.84 (m, 3H, CH, Ar), 7.70 (d, *J* = 8.1 Hz, 1H, CH, Ar), 7.51 (dd, *J* = 8.5, 1.9 Hz, 1H, CH, Ar, naproxen), 7.39–7.42 (m, 1H, CH, Ar), 7.27–7.29 (m, 2H, CH, Ar), 7.16 (dd, *J* = 8.9, 2.6 Hz, 1H, CH, Ar, naproxen), 4.21 (q, *J* = 7.1 Hz, 1H, *CH, naproxen), 3.88 (s, 3H, OCH_3_), 1.59 (d, *J* = 7.0 Hz, 3H, CH_3_, naproxen). ^13^C-NMR (151 MHz, DMSO-*d*_6_, 80 °C, δ ppm) 173.8 (C=O, amide), 157.9 (COCH_3_, Ar, naproxen), 136.3 (CH, Ar), 134.0 (CH, Ar), 132.1 (CH, Ar), 129.6 (CH, Ar), 129.0 (CH, Ar), 127.4 (CH, Ar), 126.7 (CH, Ar), 126.4 (CH, Ar), 126.3 (CH, Ar), 123.9 (CH, Ar), 121.9 (CH, Ar), 120.9 (CH, Ar), 119.1 (CH, Ar), 106.7 (CH, Ar), 55.8 (OCH_3_, naproxen), 45.6 (*CH, naproxen), 18.6 (CH_3_, naproxen). IR (KBr) ν_max_, cm^−1^: 3327 ν(N-H), 2933, 2850 ν(C_sp_^2^-H), 1700 ν(C=O), 1604 δ(N-H), 1596 ν(C=C, Ph), 1540 δ(N-H), ν(C-N), 1442 ν(C=C, Ph), 1392 δ_as_(CH_3_), δ(N-CH_2_), 1267, 1229 ν(C-N), 852, 756 γ(C_sp_^2^-H), 669 ν(C-S-C). HRMS electrospray ionization (ESI) *m/z* calcd for [M+H]^+^ C_21_H_19_N_2_O_2_S^+^ = 363.1162, found 363.1155 (mass error ∆m = −1.93 ppm).

**3d (*N*-(benzo[*d*]thiazol-2-yl)-2-(6-chloro-9H-carbazol-2-yl)propanamide)**

White crystals (m.p. 196–198 °C), yield of 71% (0.2882 g), isolated by column chromatography on silica gel with a mixture of petroleum/diethyl ether 1:1. ^1^H-NMR (600 MHz, DMSO-*d*_6_, 80 °C, δ ppm) 12.23 (brs, 1H, NH, amide), 11.17 (s, 1H, NH, carprofen), 8.11 (d, *J* = 2.1 Hz, 1H, CH, Ar), 8.08 (d, *J* = 8.1 Hz, 1H, CH, Ar), 7.92 (d, *J* = 8.7 Hz, 1H, CH, Ar), 7.70 (d, *J* = 7.5 Hz, 1H, CH, Ar), 7.54 (s, 1H, CH, Ar), 7.47 (d, *J* = 8.5 Hz, 1H, CH, Ar), 7.40 (t, *J* = 7.0 Hz, 1H, CH, Ar), 7.34 (dd, *J* = 8.7, 2.1 Hz, 1H, CH, Ar, carprofen), 7.28 (t, *J* = 7.6 Hz, 1H, CH, Ar), 7.24 (dd, *J* = 8.2, 1.6 Hz, 1H, CH, Ar, carprofen), 4.23 (q, *J* = 7.0 Hz, 1H, *CH, carprofen), 1.59 (d, *J* = 7.0 Hz, 3H, CH_3_, carprofen). ^13^C-NMR (151 MHz, DMSO-*d*_6_, 80 °C, δ ppm) 173.9 (C=O, amide), 158.6 (CH, Ar), 141.3 (CH, Ar), 139.7 (CH, Ar), 139.1 (CH, Ar), 132.1 (CH, Ar), 126.4 (CH, Ar), 125.6 (CH, Ar), 124.2 (CH, Ar), 123.9 (CH, Ar), 123.6 (CH, Ar), 121.9 (CH, Ar), 121.3 (CH, Ar), 121.0 (CH, Ar), 120.9 (CH, Ar), 120.0 (CH, Ar), 119.3 (CH, Ar), 112.8 (CH, Ar), 110.5 (CH, Ar), 46.1 (*CH, carprofen), 19.0 (CH_3_, carprofen). IR (KBr) ν_max_, cm^−1^: 3420 ν(N-H), 2973, 2933 ν(C_sp_^2^-H), 1684 ν(C=O), 1612 δ(N-H), 1599 ν(C=C, Ph), 1539 δ(N-H), ν(C-N), 1473, 1444 ν(C=C, Ph), 1337 δ_as_(CH_3_), δ(N-CH_2_), 1270, 1244 ν(C-N), 871, 755 γ(C_sp_^2^-H), 680 ν(C-S-C). HRMS electrospray ionization (ESI) *m/z* calcd for [M+H]^+^ C_22_H_17_ClN_3_OS^+^ = 406.0776, found 406.0770 (mass error ∆m = −1.23 ppm).

**3e (*N*-(benzo[*d*]thiazol-2-yl)-2-(2-fluoro-[1,1′-biphenyl]-4-yl)propanamide)**

Reported by our group in previous research [45].

### 3.3. HRMS Analysis

The HESI source was operated in positive ionization mode under the following conditions: a spray voltage of +3.5 kV, capillary and probe heater temperatures set to 320 °C, a sheath gas flow rate of 36 arbitrary units (a.u.), an auxiliary gas flow rate of 11 a.u., and a spare gas flow rate of 1 a.u. (where a.u. refers to values configured by Exactive Tune software - Q Exactive Plus - Orbitrap MS 2.8 SP1 build 2806). The S-Lens RF level was set to 50.00. Nitrogen served as both the sample nebulization gas and the collision gas within the HCD cell. For mass spectrometry analysis, 1 µL of sample solution (approximately 20 µg/mL^−1^) was introduced into the mass spectrometer via the UHPLC system. Chromatographic runs were performed isocratically using a mobile phase consisting of water, acetonitrile, methanol, and acetic acid in a 25:50:25:0.2 ratio, with a solvent flow rate of 300 µL/min^−1^. The mass spectrometry experiment employed was Full MS–ddMS^2^ (Top 5). In the full-scan MS, the instrument settings included a resolution of 70,000 (at *m/z* 200), an automatic gain control (AGC) target of 3 × 10^6^, a maximum injection time (IT) of 100 ms, and a mass range of *m/z* 100–500. For ddMS^2^ scans, the parameters were as follows: a resolution of 17,500 (at *m/z* 200), an AGC target of 1 × 10^5^, a maximum IT of 50 ms, a loop count of 5, an isolation window of 2.0 *m/z*, and stepped normalized collision energy (NCE) levels of 10, 20, and 60. The data-dependent (dd) settings included a maximum AGC target of 5 × 10^4^, a dynamic exclusion duration of 1 s, and the application of preferred peptide matching and isotope exclusion. Data acquisition and processing were carried out using Xcalibur software version 4.0 (Thermo Fisher Scientific, Waltham, MA, USA).

### 3.4. Biological Activity

#### 3.4.1. Hydrogen Peroxide Scavenging Activity (HPSA)

The Manolov et al. approach was used to evaluate the capacity to scavenge hydrogen peroxide [46]. A 43 mM solution of H_2_O_2_ was prepared in a potassium phosphate buffer solution (0.2 M, pH 7.4). The analysis of the samples was carried out as follows: in test tubes, 0.6 mL H_2_O_2_ (43 mM), 1 mL sample/standard of different concentrations (20–1000 µg/mL), and 2.4 mL potassium phosphate buffer solution were mixed. The mixture was stirred and incubated in the dark for 10 min at 37 °C. Absorbance was measured at 230 nm with a spectrophotometer (Camspec M508, Leeds, UK) against a blank solution containing phosphate buffer and H_2_O_2_ without the sample. Ascorbic acid and quercetin were used as standards. The percentage HPSA of the samples was evaluated by comparing them to a blank sample and was calculated using the following formula:

(1) $$I,\%(HPSA)=\left[\frac{A_{blank}-(A_{TS}-A_{CS})}{A_{blank}}\right]\times 100$$

where *A_blank_* is the absorbance of the blank sample, *A_CS_* is the absorbance of the control sample, and ATS is the absorbance of the test sample.

#### 3.4.2. Hydroxyl Radical Scavenging Activity (HRSA)

Hydroxyl radical scavenging activities of different fractions of 2-ABT-profenes were determined according to the method described by Guo [47]. The scavenging of hydroxyl radicals was performed as follows: 0.3 mL sodium salicylate (20 mM), 1 mL FeSO_4_ (1.5 mM), 1 mL sample/standard of different concentrations (20–1000 µg/mL), and 0.7 mL H_2_O_2_ (6 mM) were mixed immediately. Then, the reaction tubes were put in a 37 °C water bath for 1 h, and the absorbance of the mixture was recorded at 510 nm against a blank. We used standards with proven high antioxidant activity such as ascorbic acid and quercetin. The hydroxyl radical scavenging ability was calculated as follows:

(2) $$I,\%(HRSA)=\left[\frac{A_{blank}-A_{sample}}{A_{blank}}\right]\times 100$$

where *A_blank_* is the absorbance without samples and *A_sample_* is the absorbance in the presence of the samples.

#### 3.4.3. Inhibition of Albumin Denaturation (IAD)

In vitro analysis of anti-inflammatory activity was assessed as the inhibition of albumin denaturation (IAD). The analysis was performed according to Manolov’s method [48], with minor modifications. The experiment was performed with human albumin. The solution of albumin (1%) was prepared in distilled water (pH 7.4). Test samples/standards were first dissolved in 1.2 mL of DMSO and then supplemented with 1% Tween 80 in PBS so that the final concentration of the stock solution was 1000 μg/mL. Then, a series of working solutions with different concentrations (20–500 μg/mL) in 1% Tween 80/PBS were prepared. The reaction mixture contained a 2 mL test sample/standard of different concentrations and 1 mL albumin (1%). The mixture was incubated at 37 °C for 15 min and then heated at 70 °C for 15 min in a water bath. After cooling, the turbidity was measured at 660 nm with a spectrophotometer (Camspec M508, Leeds, UK). Ibuprofen was used as a standard. The experiment was performed three times. The percentage inhibition of albumin denaturation (IAD) was calculated against the control. The control sample consisted of the same concentration of albumin dissolved in distilled water.

(3) $$\%IAD=\left[\frac{A_{blank}-A_{sample}}{A_{blank}}\right]\times 100$$

#### 3.4.4. Molecular Docking

The molecular docking study of ligands **3a**–**e** was conducted against human serum albumin (HSA) using AutoDock Vina 1.1.2 (ADV) and AutoDock 4.2 (AD) [49,50]. The HSA structure was obtained from the Protein Data Bank (PDB ID: 7JWN) [51]. ADV and AD are based on distinct computational principles, providing complementary approaches to reduce the inherent false positives of molecular docking [52]. Ligands were prepared as both R and S isomers using Avogadro 1.2.0 for each compound, following established protocols [52].

The preparation of HSA was made using the standard procedures previously reported by our group, including co-crystallized ligand removal, addition of polar hydrogen atoms, and charge assignment [53,54]. Final file preparations for both ligands and the macromolecule were completed using AutoDockTools 1.5.6 [50]. The molecular docking targeted four primary binding sites on HSA: Sudlow site I (subdomain IIA) and II (subdomain IIIA), site III, and the cleft region. These sites were chosen due to their prominent roles in drug binding, as reported in the literature, while other identified sites are considered less critical for drug interactions with albumin [55,56,57,58,59,60]. The search space for each binding site was defined as a cube with dimensions of 20 Å for ADV and 54 for AD (spacing = 0.375). The coordinates for the center of each site were as follows: Sudlow I (x = 30.62, y = 25.50, z = 12.43), Sudlow II (x = 5.95, y = 18.22, z = 21.06), site III (x = 30.15, y = 26.98, z = 37.99), and the cleft site (x = 20.89, y = 21.74, z = 22.43) [54].

To increase the reproducibility of the results, ADV was configured to generate 20 poses per ligand per targeted site, while AD was set to generate 200 poses per targeted site to support clustering analysis using a 2 Å root mean square deviation (RMSD) threshold. Finally, the docking results were visualized using Chimera 1.10.2 [61].

#### 3.4.5. Molecular Dynamics Simulations

For each binding site, the top binding compound was used for the construction of a chimeric complex with HSA, with the respective ligand bound, and the temporal stability of the respective ligand–albumin complexes was assessed by molecular dynamics simulations. The simulations were performed using GROMACS 2023 with the CHARMM36 force field and the TIP4P water model within an orthorhombic simulation box [62,63,64]. The simulations ran on a system with Debian 11, an Intel Core i7-12700KF CPU (Santa Clara, CA, USA), and an NVIDIA RTX 3060 GPU (Santa Clara, CA, USA), utilizing CUDA 12 for GPU acceleration. Ligand parameterization was carried out via the CgenFF, and the systems were constructed, neutralized, and relaxed through energy minimization, as outlined in previous studies [65,66,67]. The simulations ran for 100 ns, and the time-dependent behavior of the ligand–albumin complexes was visualized with VMD 1.9.4 [68].

#### 3.4.6. Determination of Lipophilicity as RM Values

The determination of lipophilicity of 2-ABT derivatives was estimated according to the method reported by Pontiki and Hadjipavlou-Litina [43].

#### 3.4.7. Statistical Analysis

All the analyses were made in triplicates. The data in this paper are expressed as mean ± SD. The level of significance was set to *p* < 0.05. SPSS 19.0 software was used for data analysis by one-way ANOVA followed by Duncan’s post hoc test to evaluate differences between mean values of activities (SPSS Inc., Chicago, IL, USA).

## 4. Conclusions

An efficient approach for the synthesis of four novel benzothiazole–profen amides was identified and presented in the current paper. The applied reaction offers a convenient synthetic route for coupling benzothiazoline moiety to various profens. The bioprofile of the newly obtained hybrids gives a good lead for their use as potential NSAIDs. Antioxidant activity is closely linked to anti-inflammatory activity as oxidative stress frequently contributes to the development of inflammatory processes. High antioxidant activity can help suppress the oxidative mechanisms that drive inflammation. Consequently, 2-ABT derivatives with strong antioxidant properties, such as **3b** (HPSA IC_50_ = 60.24 μg/mL) and **3c** (HPSA IC_50_ = 67.71 μg/mL), also demonstrated notable anti-inflammatory activity, as indicated by their IAD measurements (**3b** = 54.64 μg/mL and **3c** = 64.44 μg/mL). Molecular docking studies further supported these findings, revealing favorable binding interactions between the hybrid compounds and key targets involved in inflammation and oxidative stress. The docking results indicate that these benzothiazole–profen hybrids could effectively modulate biological pathways associated with inflammation, enhancing their therapeutic potential. Furthermore, the in silico study identified the ***S*** isomer of **3b** as the most active compound, reinforcing its potential. These findings highlight **3b** as a leading candidate with potent antioxidant and anti-inflammatory properties, balanced lipophilicity, and strong pharmacological potential.

## Figures and Tables

**Table 1:** The results of the molecular docking study conducted with AutoDock Vina, presented as the binding affinity of the studied compounds to the four targeted HSA sites, are expressed in terms of variation in Gibbs free energy (ΔG kcal/mol).

Compound	Isomer	Sudlow I	Sudlow II	Site III	Cleft
**3a**	R	−8.7	−10.0	−10.3	−9.6
	S	−8.4	−10.6	−10.4	−9.6
**3b**	R	−9.9	−11.8	−12.2	−11.0
	S	−9.8	−12.0	−11.9	−11.1
**3c**	R	−9.1	−11.1	−11.5	−10.0
	S	−9.0	−11.4	−12.0	−10.1
**3d**	R	−9.5	−11.1	−11.1	−11.0
	S	−9.5	−11.8	−11.3	−10.7
**3e**	R	−9.0	−11.5	−11.4	−10.5
	S	−9.1	−12.3	−11.8	−11.2

**Table 2:** The results of the molecular docking study conducted with AutoDock, presented as the binding affinity of the studied compounds to the four targeted HSA sites, are expressed in terms of variation in Gibbs free energy (ΔG kcal/mol) and the cluster analysis of the conformations.

Compound	Isomer	Sudlow I		Sudlow II		Site III		Cleft	
		ΔG	%C	ΔG	%C	ΔG	%C	ΔG	%C
**3a**	R	−8.91	38	−9.73	38	−10.97	34	−8.83	50
	S	−8.53	46	−9.83	72	−10.53	46	−9.00	54
**3b**	R	−8.68	50	−11.25	32	−11.94	48	−9.82	34
	S	−8.61	32	−11.36	30	−11.92	56	−10.03	70
**3c**	R	−8.61	16	−10.51	82	−10.93	42	−9.18	28
	S	−8.70	26	−10.53	98	−10.89	42	−9.09	48
**3d**	R	−8.69	20	−11.24	84	−11.44	34	−10.02	62
	S	−8.89	56	−11.20	100	−11.50	42	−10.13	48
**3e**	R	−8.70	36	−11.42	48	−10.93	60	−9.97	50
	S	−8.88	24	−11.57	100	−11.30	84	−9.96	64

%C: percent of conformations in the same 2Å RMSD cluster of atom coordinates.

**Table 3:** The root mean square deviation of the heavy atoms of the ligands from the systems evaluated in the molecular dynamics study (nm).

System Evaluated	Sudlow I	Sudlow II	Site III	Cleft
**apo + 3e[S]**	-	0.15	-	-
**apo + 3b[R]**	0.21	-	-	-
**apo + 3b[R]**	-	-	0.33	-
**apo + 3e[S]**	-	-	-	0.27

- not tested.

**Table 4:** The root mean square deviation of the backbone of the protein from the systems evaluated in the molecular dynamics study (nm).

System Evaluated	Sudlow I	Sudlow II	Site III	Cleft
**apo + 3e[S]**	-	0.33	-	-
**apo + 3b[R]**	0.29	-	-	-
**apo + 3b[R]**	-	-	0.24	-
**apo + 3e[S]**	-	-	-	0.35
**apo**	0.38			

- not tested.

**Table 5:** The radius of gyration of the protein from the systems evaluated in the molecular dynamics study (nm).

System Evaluated	Sudlow I	Sudlow II	Site III	Cleft
**apo + 3e[S]**	-	2.77	-	-
**apo + 3b[R]**	2.78	-	-	-
**apo + 3b[R]**	-	-	2.80	-
**apo + 3e[S]**	-	-	-	2.77
**apo**	2.78			

- not tested.

**Table 6:** The average number of hydrogen bonds between the ligand and the protein in the systems evaluated in the molecular dynamics study (no/ns).

System Evaluated	Sudlow I	Sudlow II	Site III	Cleft
**apo + 3e[S]**	-	0.09	-	-
**apo + 3b[R]**	0.01	-	-	-
**apo + 3b[R]**	-	-	0.06	-
**apo + 3e[S]**	-	-	-	0.53

- not tested.

## Data Availability

Data are contained within the article and Supplementary Materials.

## Supplementary Materials

The following supporting information can be downloaded at: https://www.mdpi.com/article/10.3390/molecules30010107/s1, Figure S1: ^1^H-NMR spectrum of compound **3a**; Figure S2: ^1^H-NMR spectrum of compound **3b**; Figure S3: ^1^H-NMR spectrum of compound **3c**; Figure S4: ^1^H-NMR spectrum of compound **3d**; Figure S5: ^13^C-NMR spectrum of compound **3a**; Figure S6: ^13^C-NMR spectrum of compound **3b**; Figure S7: ^13^C-NMR spectrum of compound **3c**; Figure S8: ^13^C-NMR spectrum of compound **3d**; Figure S9: FT-IR spectrum of compound **3a**; Figure S10: FT-IR spectrum of compound **3b**; Figure S11: FT-IR spectrum of compound **3c**; Figure S12: FT-IR spectrum of compound **3d**; Figure S13: ESI-HRMS of compound **3a**; Figure S14: Mass spectrum of **3a** obtained by positive ion ESI-MS/MS; Figure S15: Proposed fragmentation of protonated **3a**; Figure S16: ESI-HRMS of compound **3b**; Figure S17: Mass spectrum of **3b** obtained by positive ion ESI-MS/MS; Figure S18: Proposed fragmentation of protonated **3b**; Figure S19: ESI-HRMS of compound **3c**; Figure S20: Mass spectrum of **3c** obtained by positive ion ESI-MS/MS; Figure S21: Proposed fragmentation of protonated **3c**; Figure S22: ESI-HRMS of compound **3d**; Figure S23: Mass spectrum of **3d** obtained by positive ion ESI-MS/MS; Figure S24: Proposed fragmentation of protonated **3d**; Figure S25: Stability during the molecular dynamics study of the complex of **3b[*R*]** docked in Sudlow’s site I of HSA; Figure S26: Stability during the molecular dynamics study of the complex of **3e[*S*]** docked in Sudlow’s site II of HSA; Figure S27: Stability during the molecular dynamics study of the complex of **3b[*R*]** docked in site III of HSA. Figure S28: Stability during the molecular dynamics study of the complex of **3e[*S*]** docked in the cleft site of HSA. Table S1: In vitro outcomes of the performed biological assays.
