# Supplementary material for: Synthesis of Novel Benzothiazole–Profen Hybrid Amides as Potential NSAID Candidates
Source: Molecules. 2024 Dec 30;30(1):107. doi: 10.3390/molecules30010107 (PMC11721736; doi:10.3390/molecules30010107)
Supplement: Supplementary file 1 [file molecules-30-00107-s001.zip › molecules-3374335-supplementary.pdf]

Supplementary Materials:

Synthesis of Novel Benzothiazole–Profen Hybrid Amides as Potential NSAID Candidates

Iliyan Ivanov [1,*], Stanimir Manolov [1], Dimitar Bojilov [1], Yordan Stremski [1], Gabriel Marc [2], Stela Statkova-Abeghe [1], Smaranda Oniga [3], Ovidiu Oniga [4] and Paraskev Nedialkov [5]

Table of Contents:

Figure S1. ^{1}H-NMR spectrum of compound **3a** page 3

Figure S2. ^{1}H-NMR spectrum of compound **3b** page 4

Figure S3. ^{1}H-NMR spectrum of compound **3c** page 5

Figure S4. ^{1}H-NMR spectrum of compound **3d** page 6

Figure S5. ^{13}C-NMR spectrum of compound **3a** page 7

Figure S6. ^{13}C-NMR spectrum of compound **3b** page 8

Figure S7. ^{13}C-NMR spectrum of compound **3c** page 9

Figure S8. ^{13}C-NMR spectrum of compound **3c** page 10

Figure S9. IR spectrum of compound **3a** page 11

Figure S10. IR spectrum of compound **3b** page 12

Figure S11. IR spectrum of compound **3c** page 13

Figure S12. IR spectrum of compound **3d** page 14

Figure S13. ESI-HRMS of compound **3a** page 15

Figure S14. Mass spectrum of **3a** obtained by positive ion ESI-MS/MS page 16

Figure S15. Proposed fragmentation of protonated **3a** page 17

Figure S16. ESI-HRMS of compound **3b** page 18

Figure S17. Mass spectrum of **3b** obtained by positive ion ESI-MS/MS page 19

Figure S18. Proposed fragmentation of protonated **3b** page 20

Figure S19. ESI-HRMS of compound **3c** page 21

Figure S20. Mass spectrum of **3c** obtained by positive ion ESI-MS/MS page 22

Figure S21. Proposed fragmentation of protonated **3c** page 23

Figure S22. ESI-HRMS of compound **3d** page 24

Figure S23. Mass spectrum of **3d** obtained by positive ion ESI-MS/MS page 25

Figure S24. Proposed fragmentation of protonated **3d** page 26

Figure S25. Stability during the molecular dynamics study of the complex of **3b[R]** docked in Sudlow's site I of HSA. page 27

Figure S26. Stability during the molecular dynamics study of the complex of **3e[S]** docked in Sudlow's site II of HSA. page 28

Figure S27. Stability during the molecular dynamics study of the complex of **3b[R]** docked in site III of HSA. page 29

Figure S28. Stability during the molecular dynamics study of the complex of **3e[S]** docked in the cleft site of HSA. page 30

Table S1. *In vitro* outcomes of the performed biological assays. The results for antioxidant activity (HPSA), Hydroxyl radical scavenging activity (HRSA), and inhibition of albumin denaturation (IAD) are expressed in terms of IC_{50} values. Ascorbic acid (AA), quercetin (Qrc), and ibuprofen (Ibu) were used as reference compounds. R_M, denoting lipophilicity, is a dimensionless measure derived from thin-layer chromatography and is dependent on the R_f value. page 31

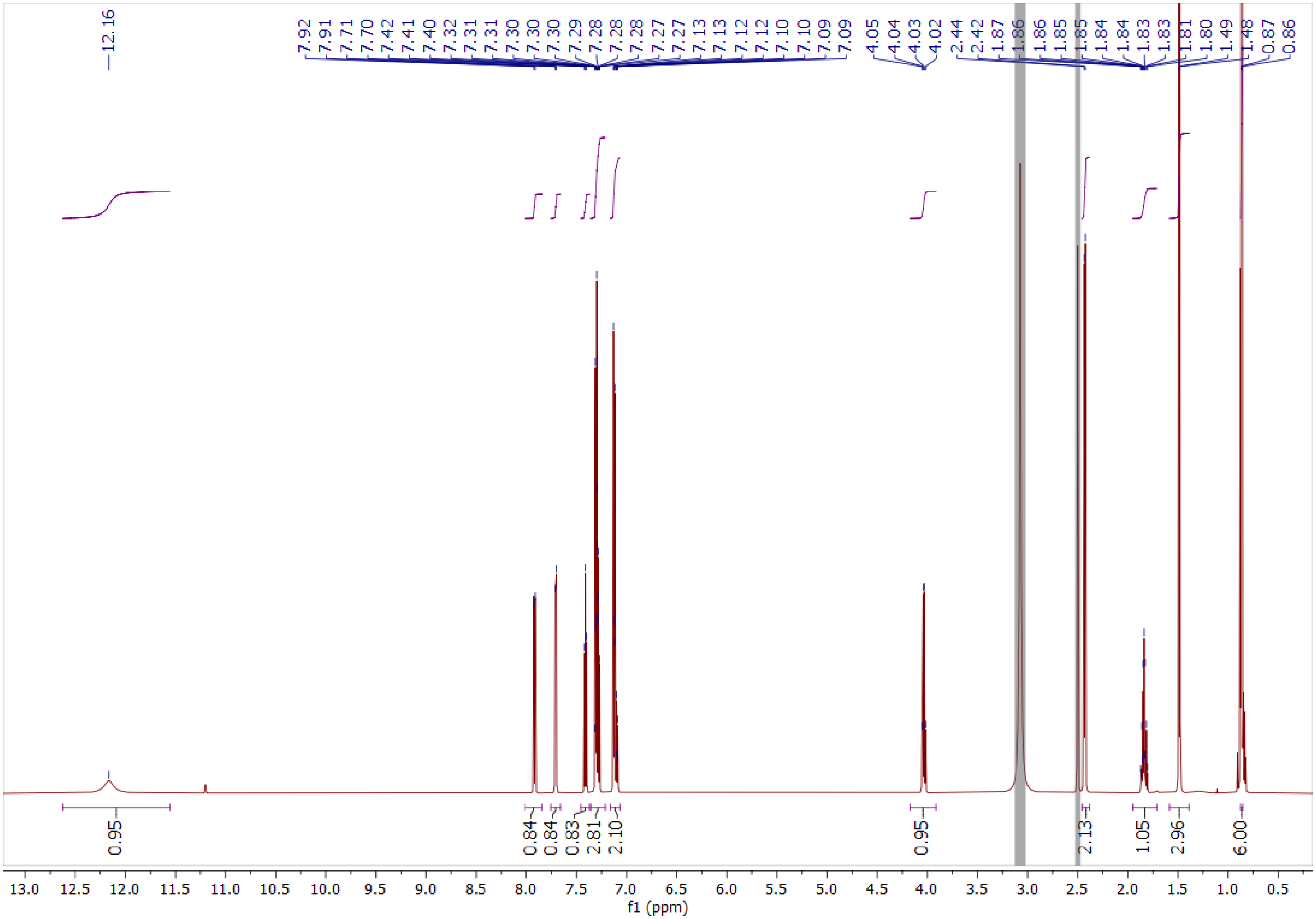

Figure S1. ^{1}H-NMR spectrum of compound **3a**.

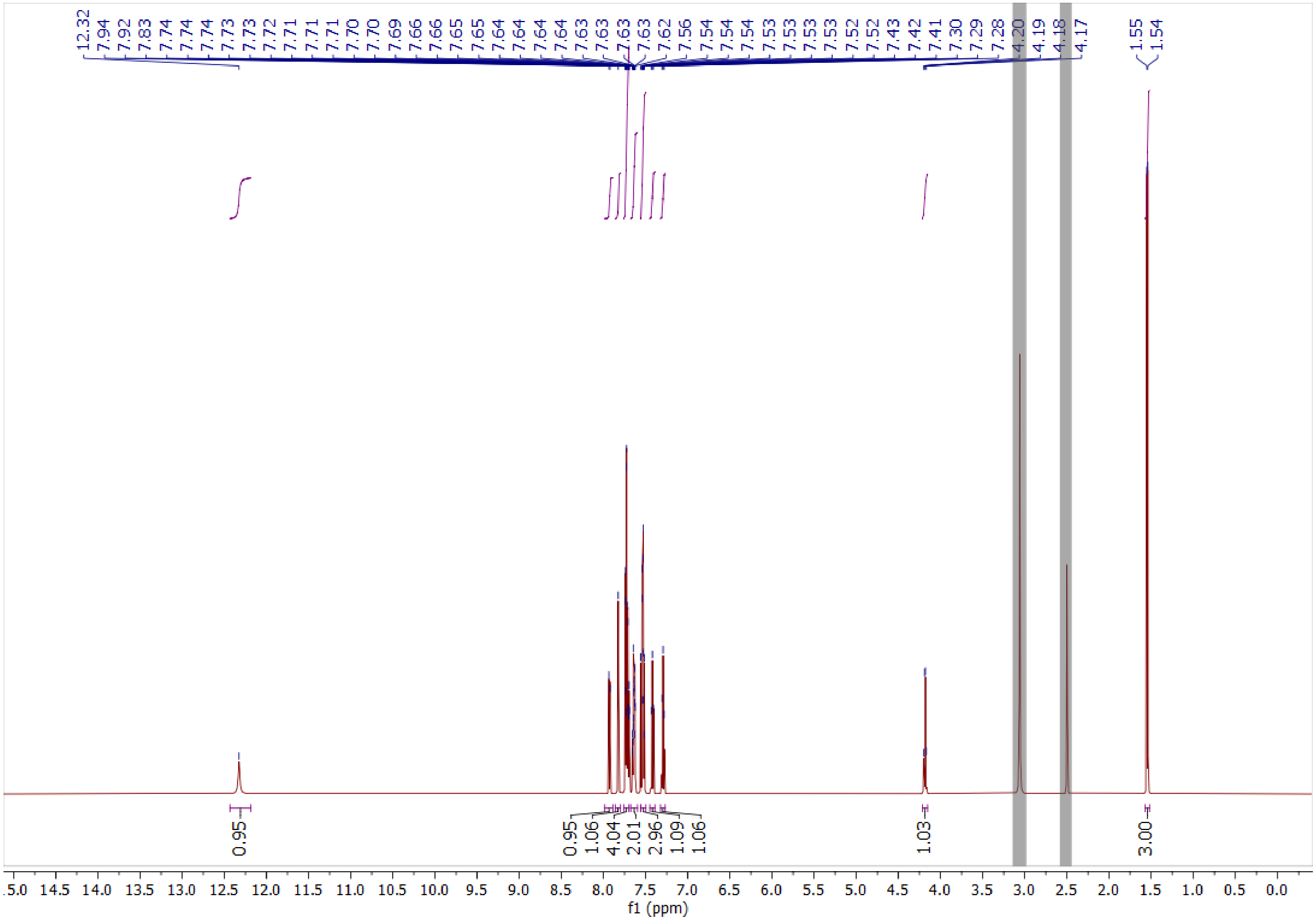

Figure S2. ^{1}H-NMR spectrum of compound **3b**.

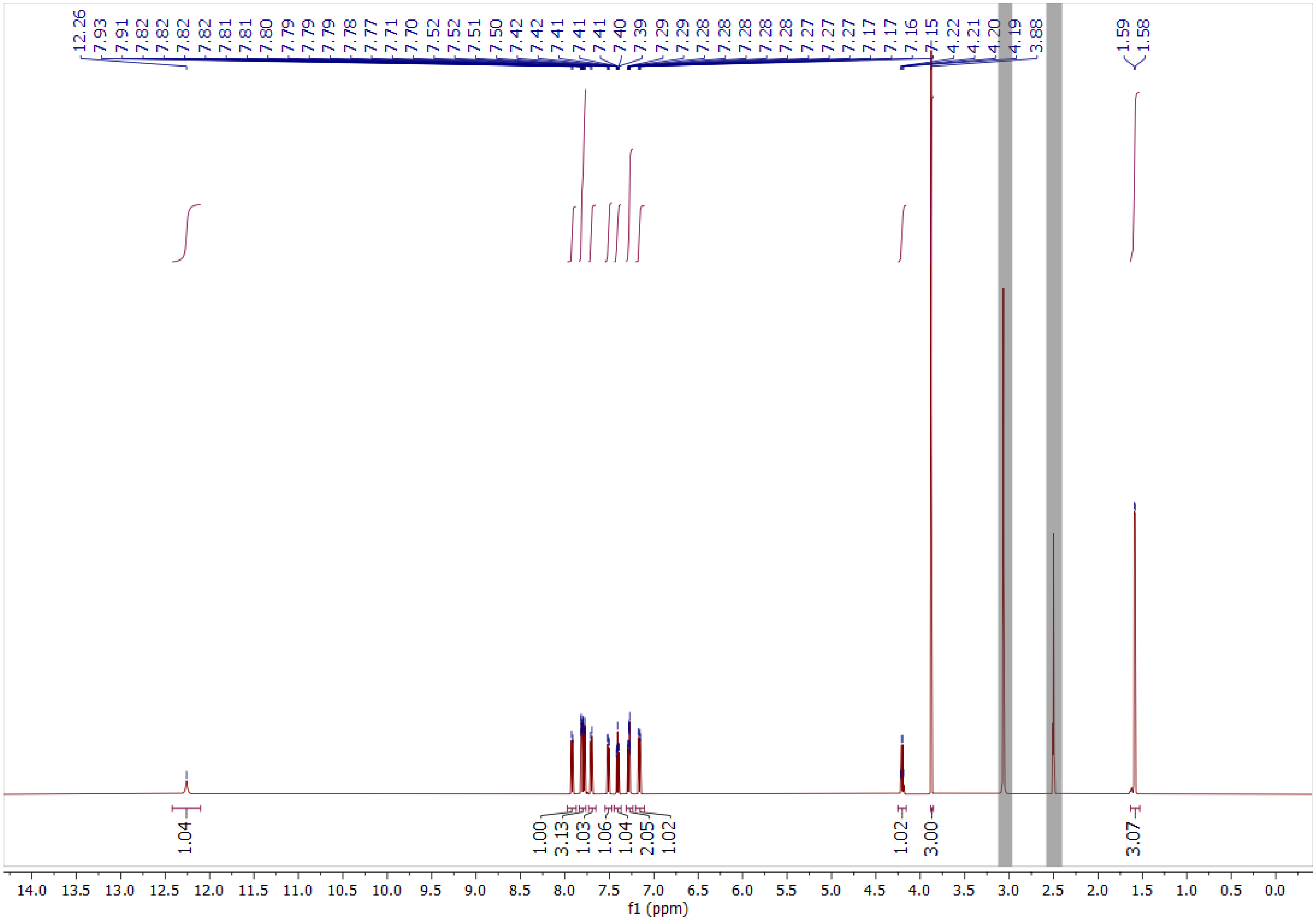

Figure S3. ^{1}H-NMR spectrum of compound **3c**.

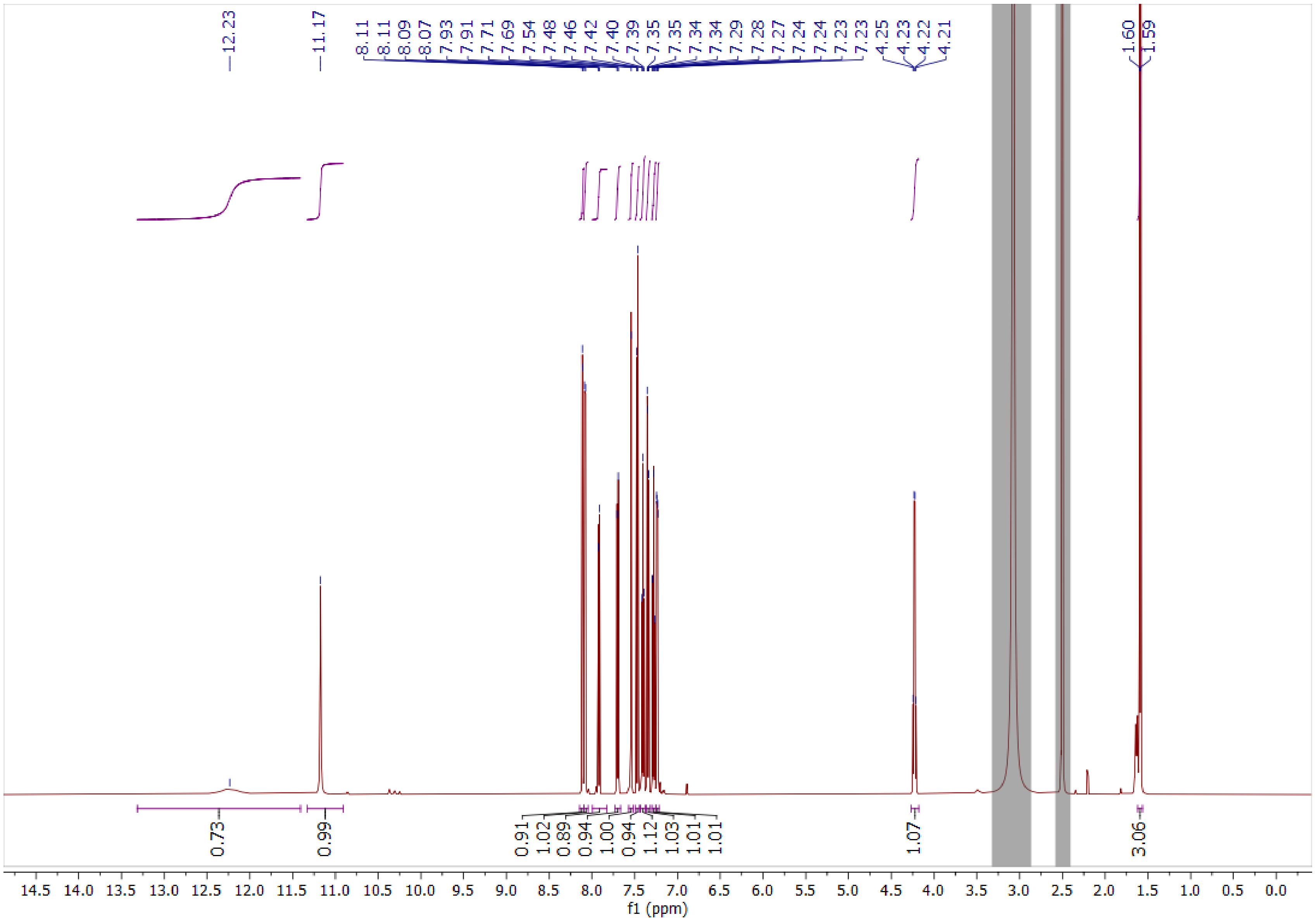

Figure S4. ^{1}H-NMR spectrum of compound **3d**.

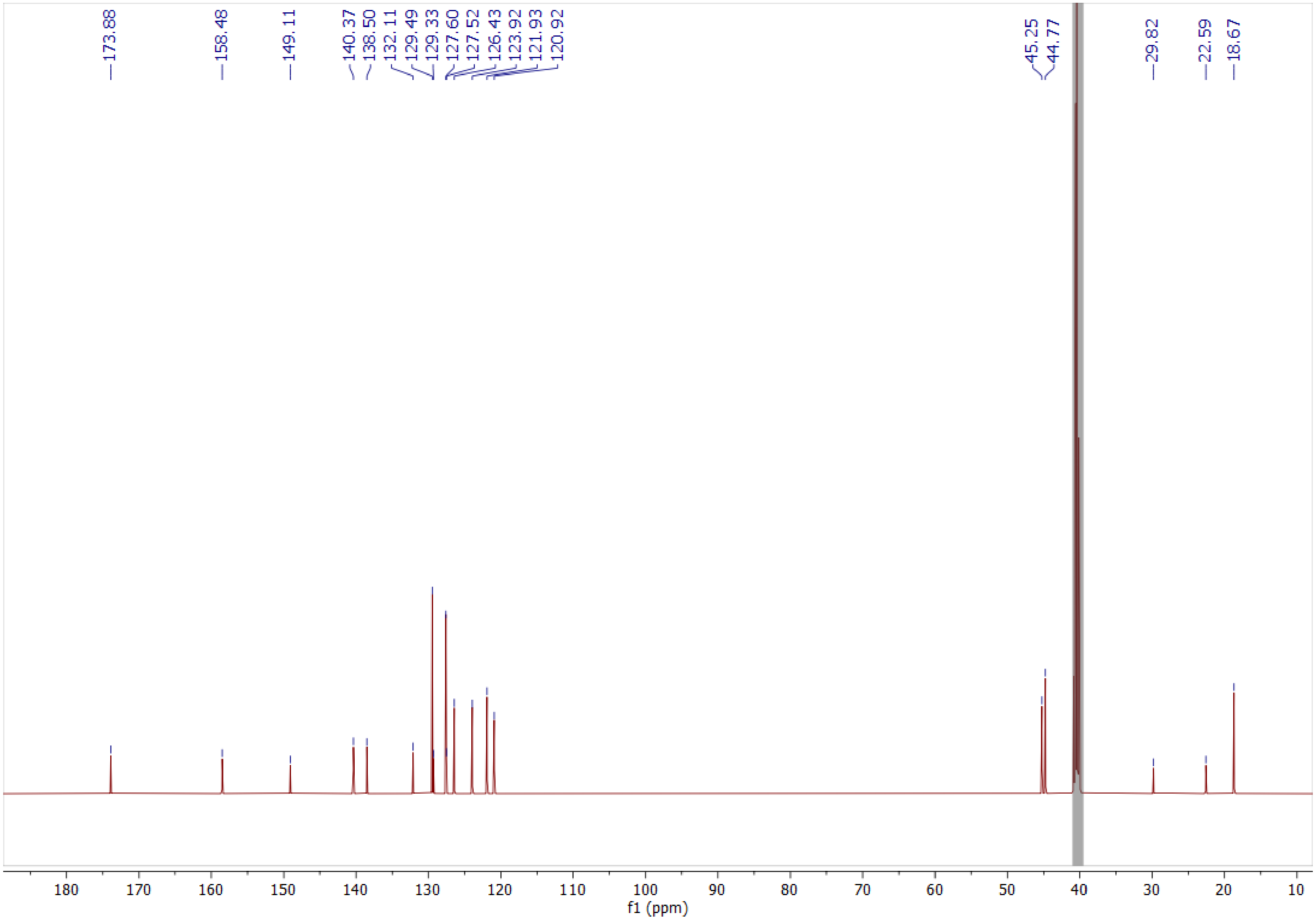

Figure S5. ^{13}C-NMR spectrum of compound **3a**.

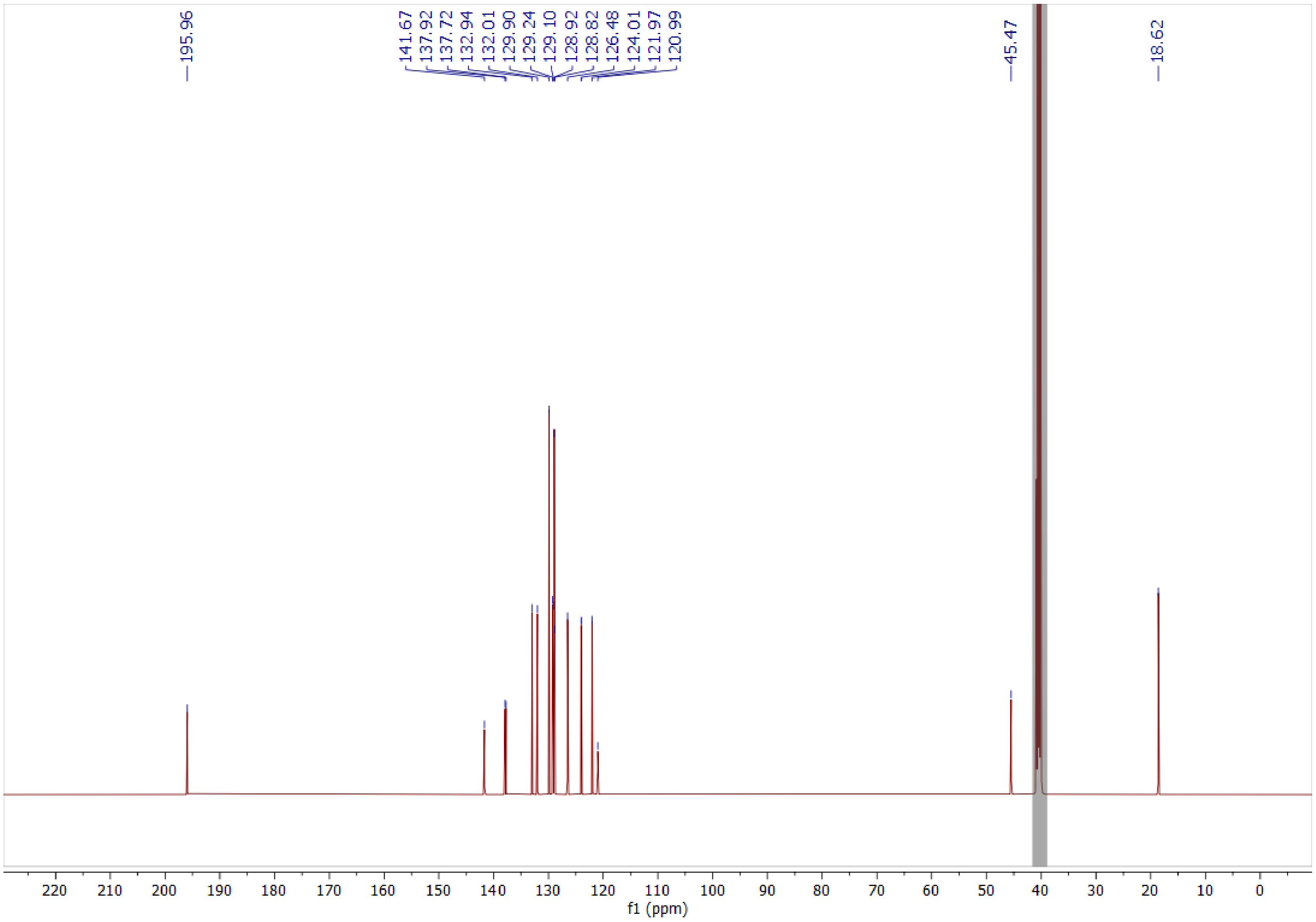

Figure S6. ^{13}C-NMR spectrum of compound **3b**.

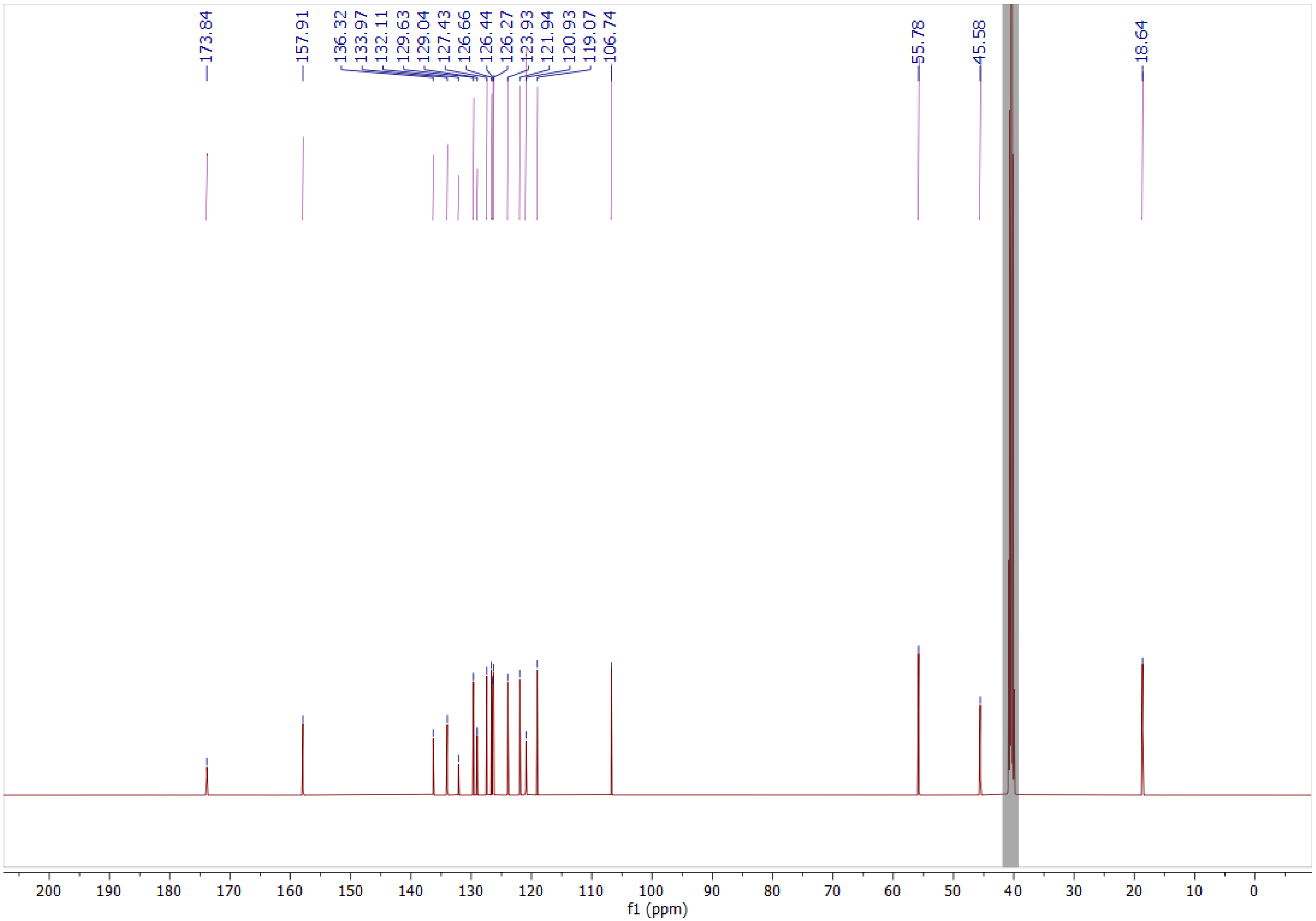

Figure S7. ^{13}C-NMR spectrum of compound **3c**.

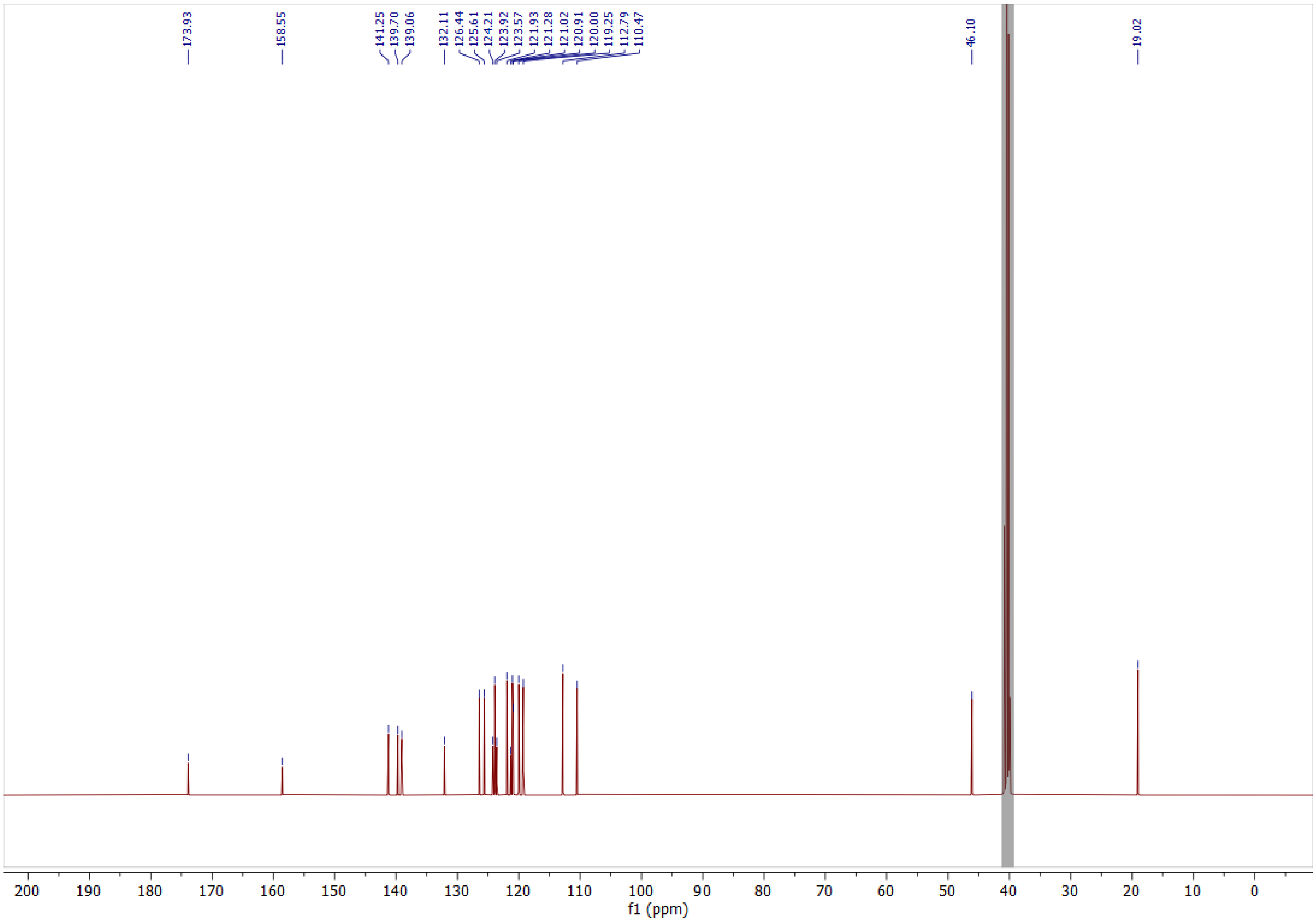

Figure S8. ^{13}C-NMR spectrum of compound **3d**.

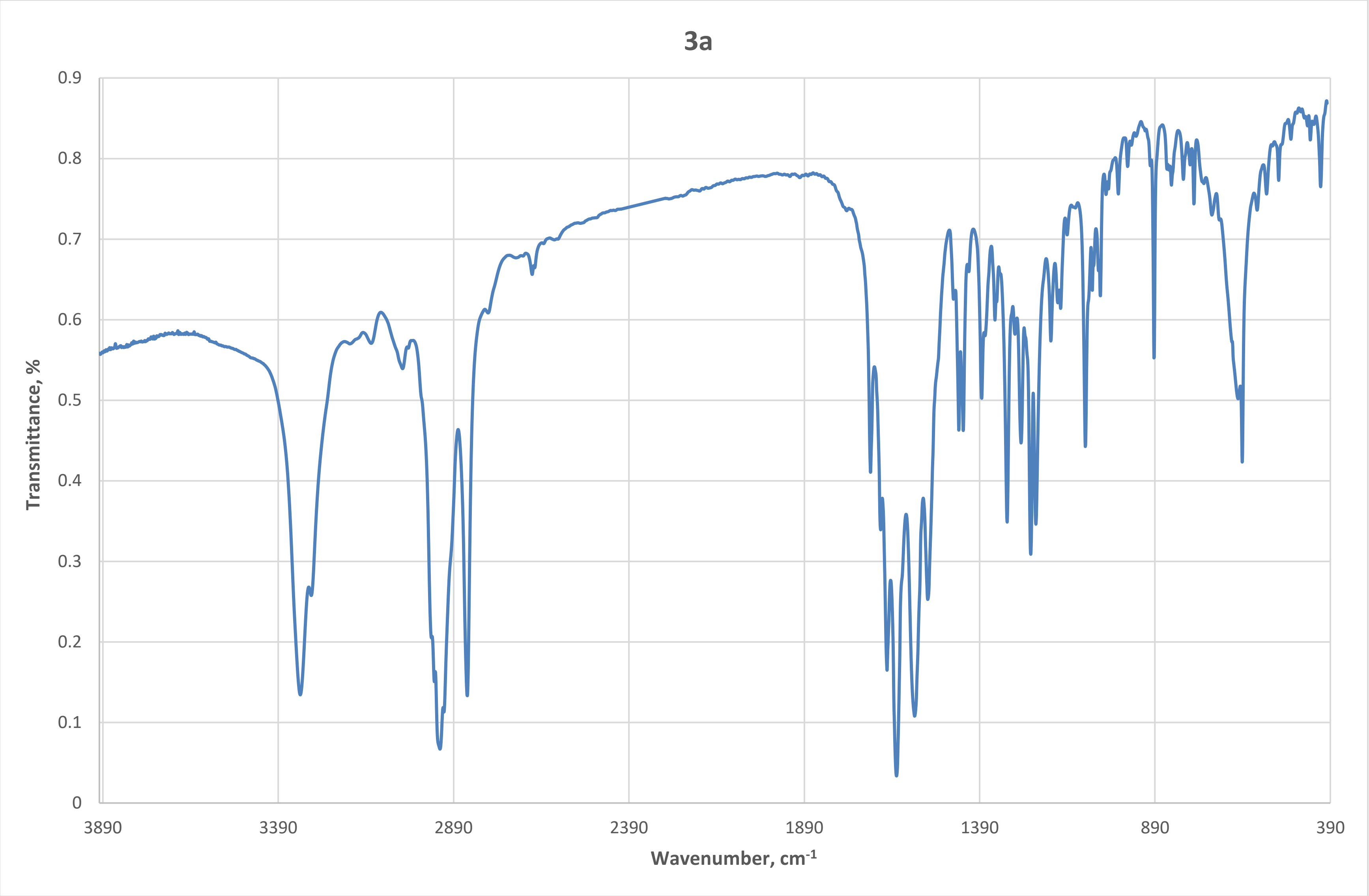

Figure S9. FT-IR spectrum of compound **3a**.

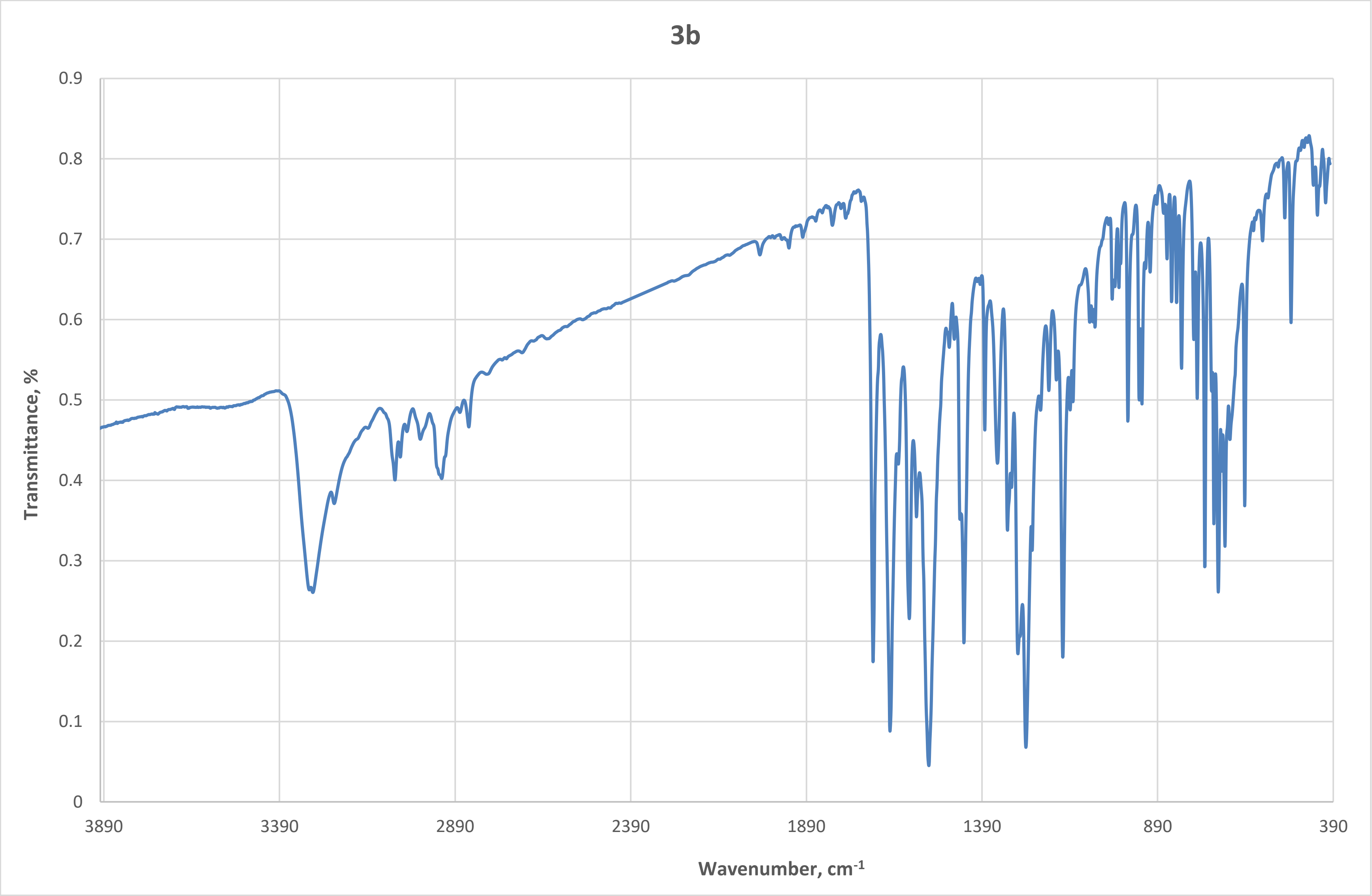

Figure S10. FT-IR spectrum of compound **3b**.

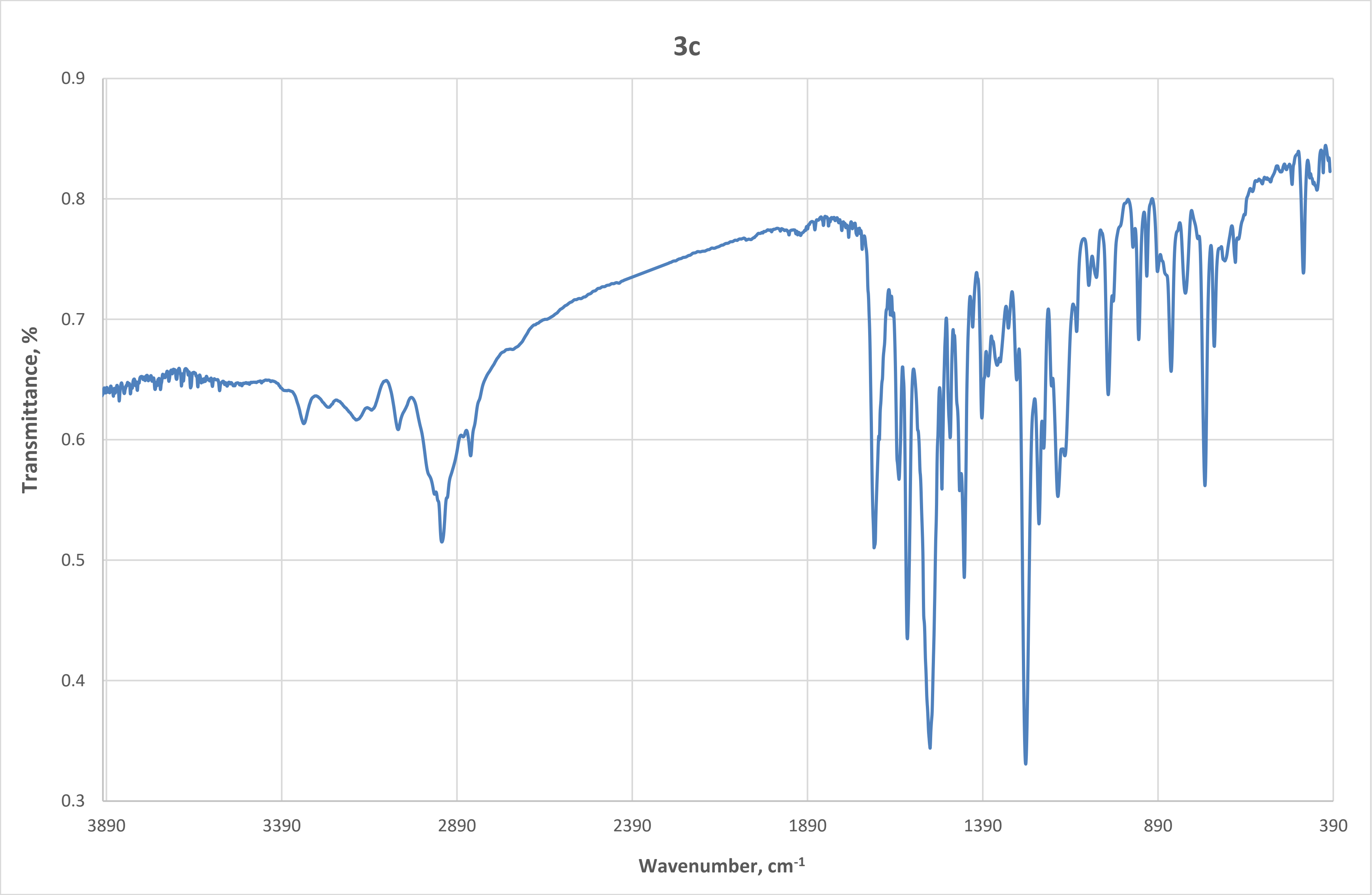

Figure S11. FT-IR spectrum of compound **3c**.

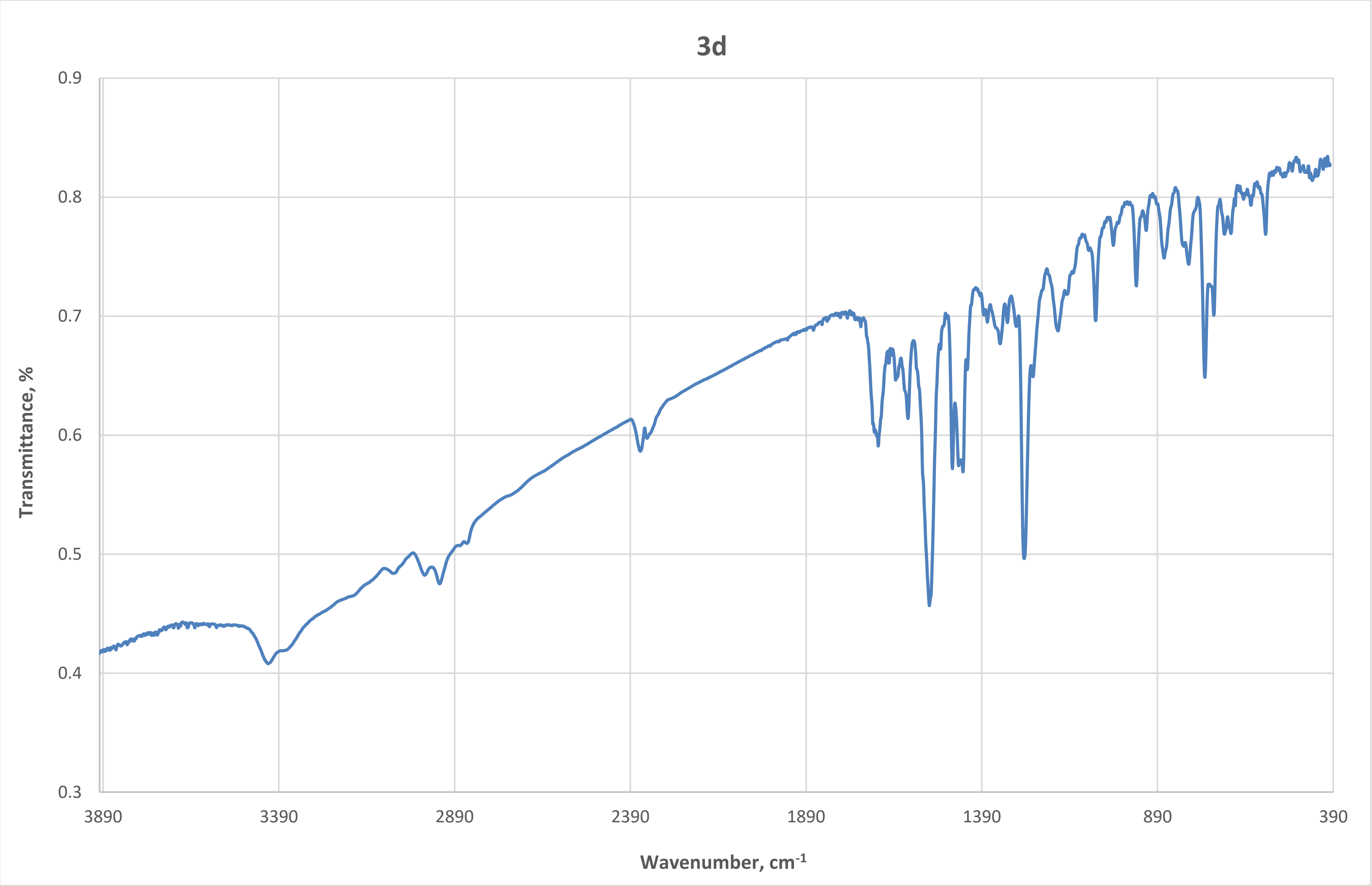

Figure S12. FT-IR spectrum of compound **3d**.

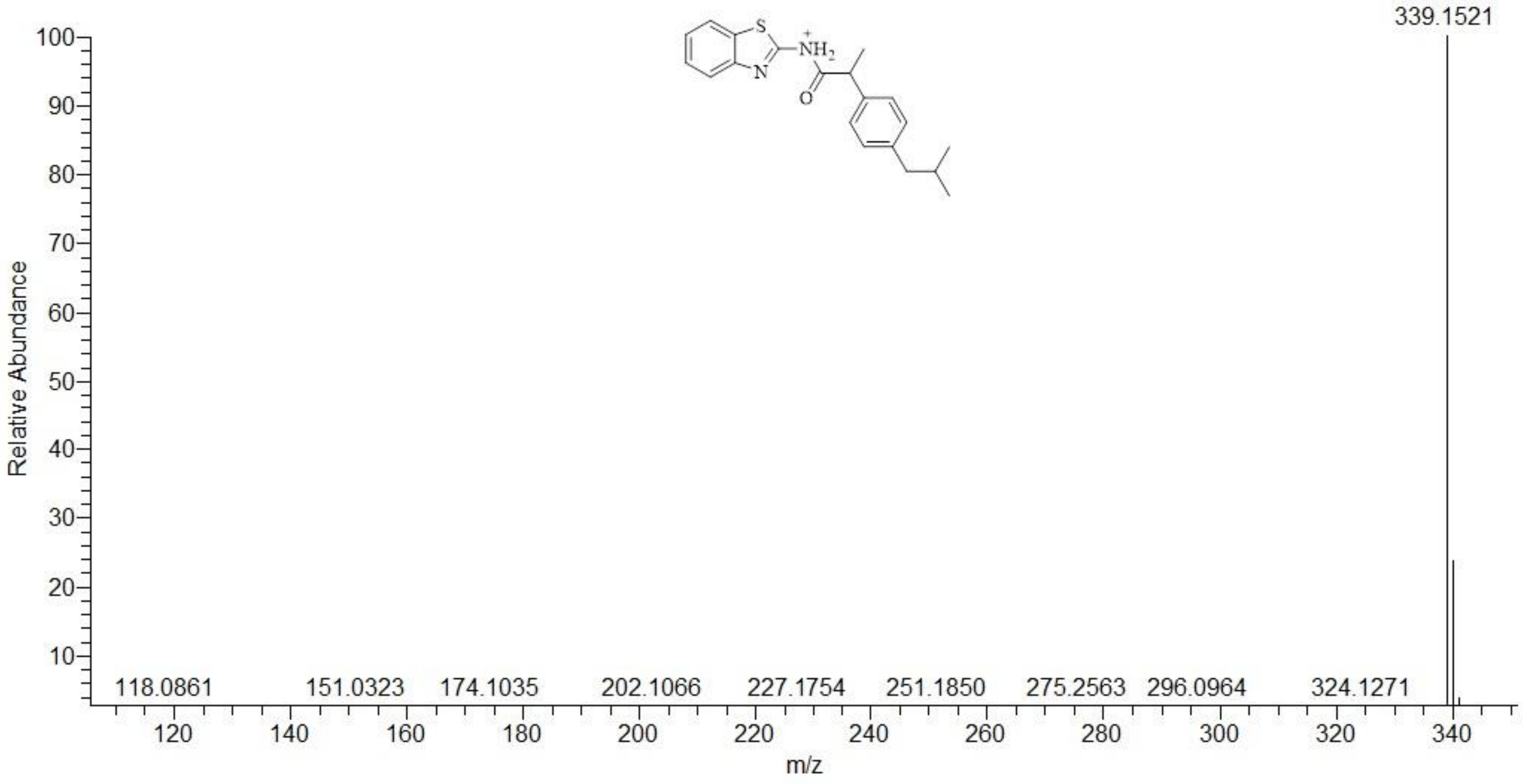

Figure S13. ESI-HRMS of compound **3a**.

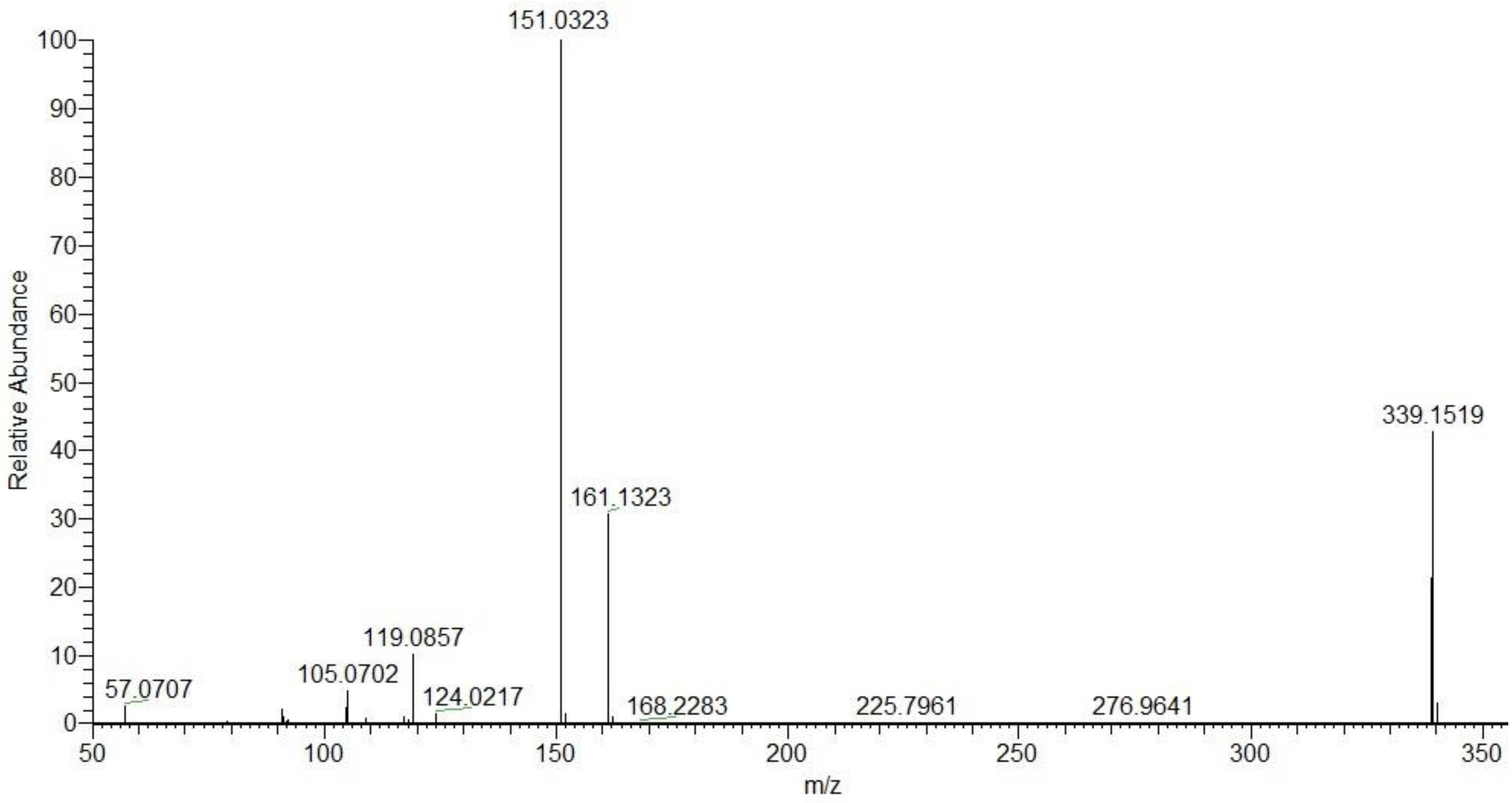

Figure S14. Mass spectrum of **3a** obtained by positive ion ESI-MS/MS.

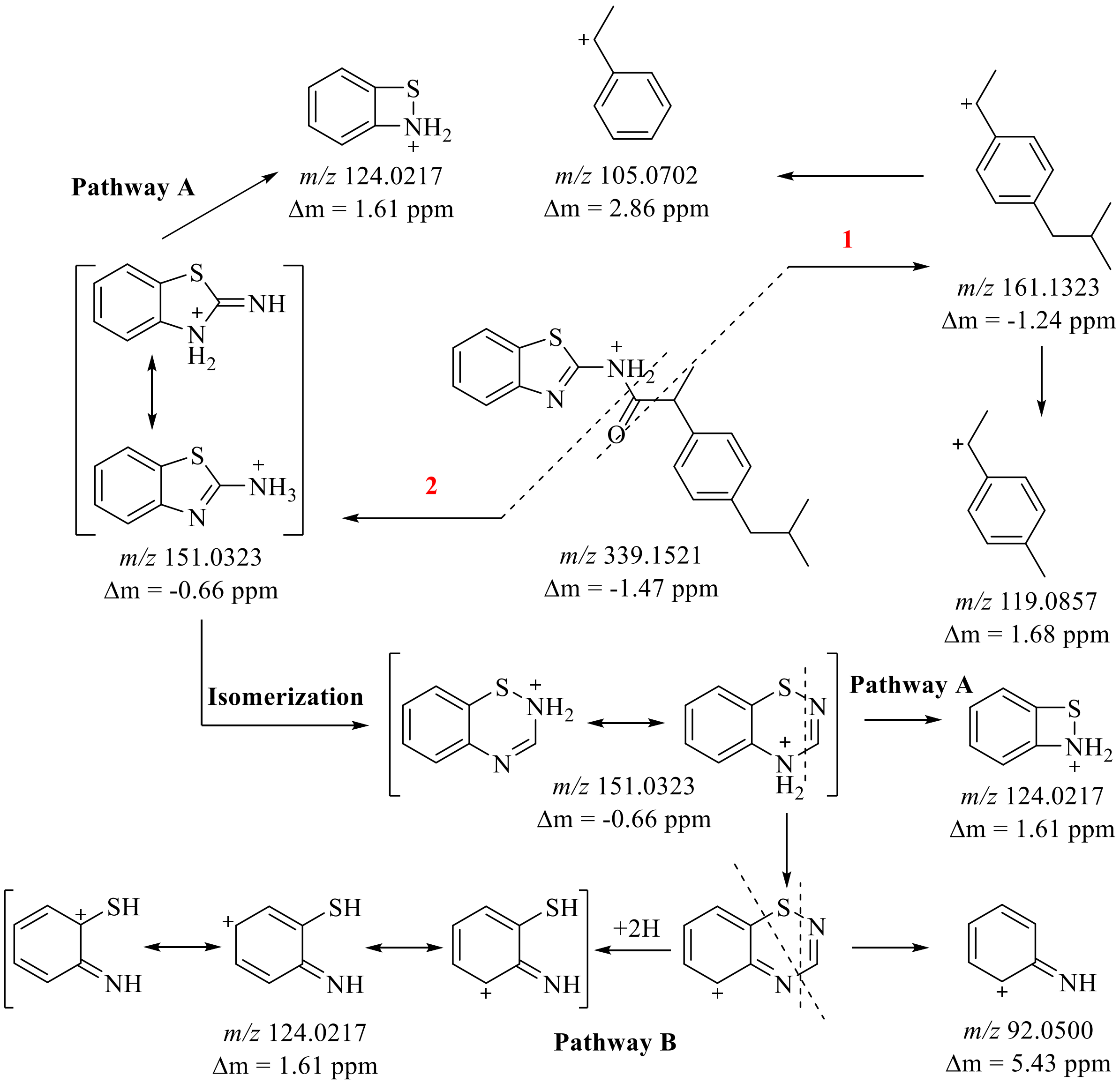

Figure S15. Proposed fragmentation of protonated **3a**.

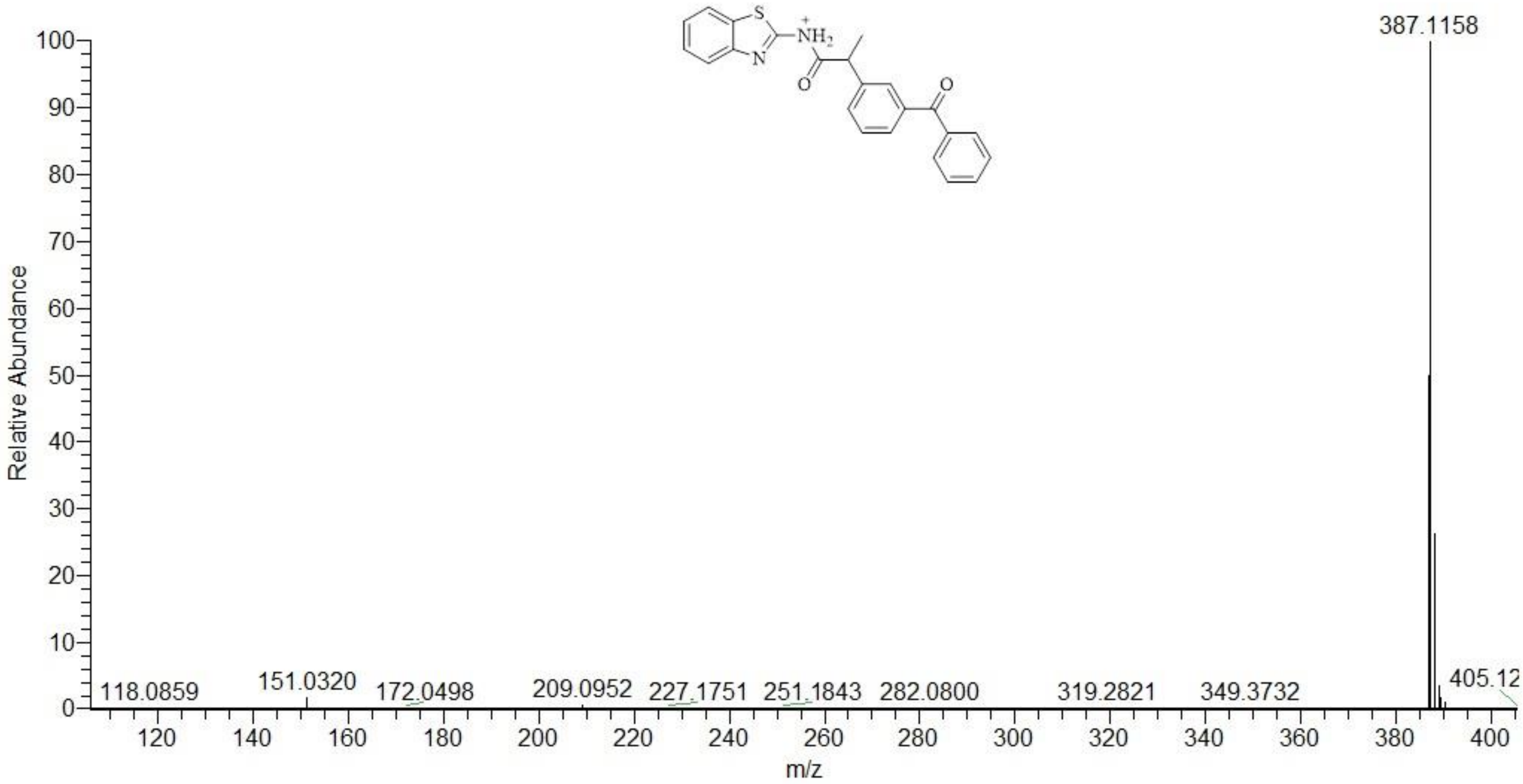

Figure S16. ESI-HRMS of compound **3b**.

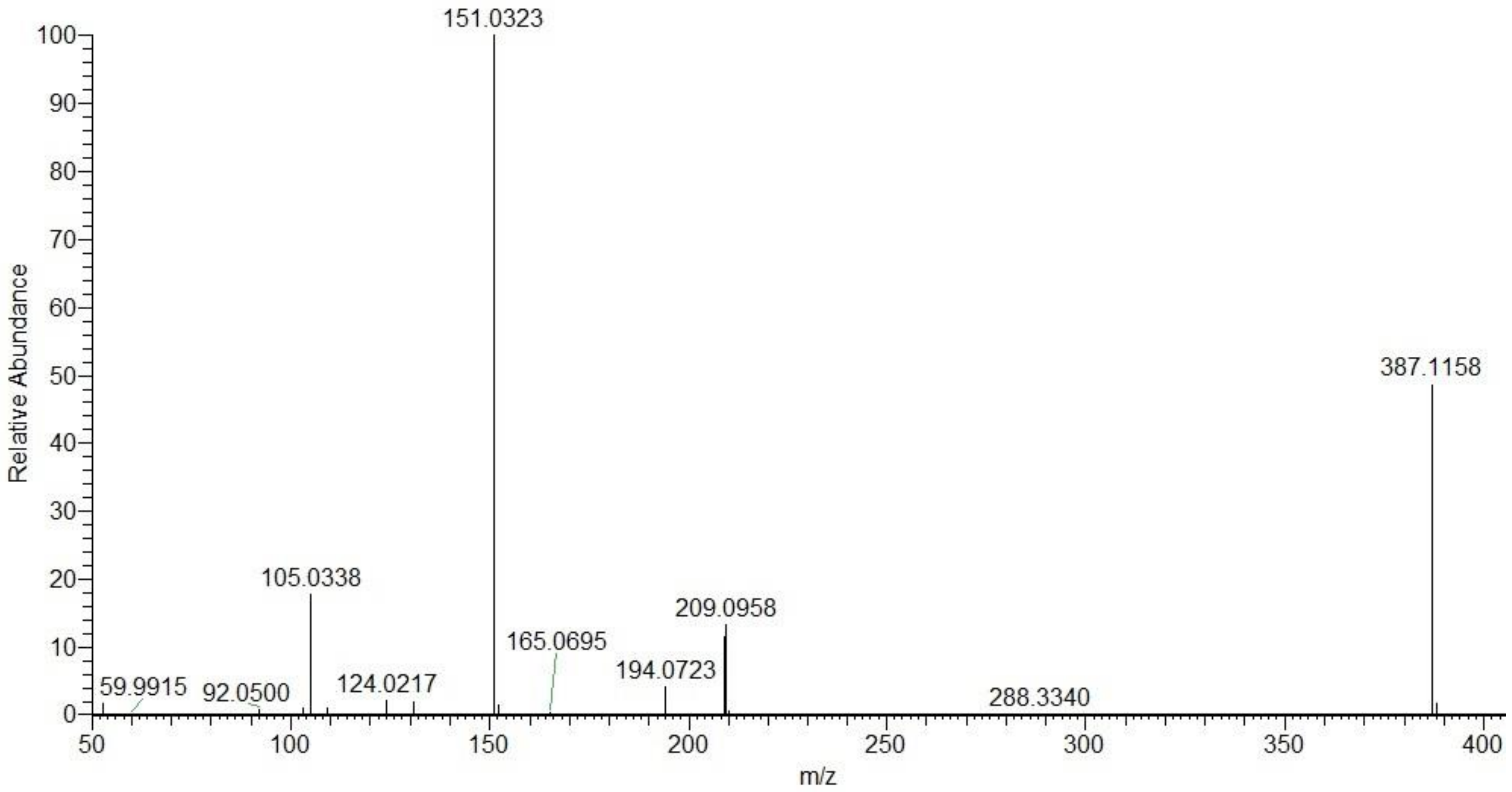

Figure S17. Mass spectrum of **3b** obtained by positive ion ESI-MS/MS.

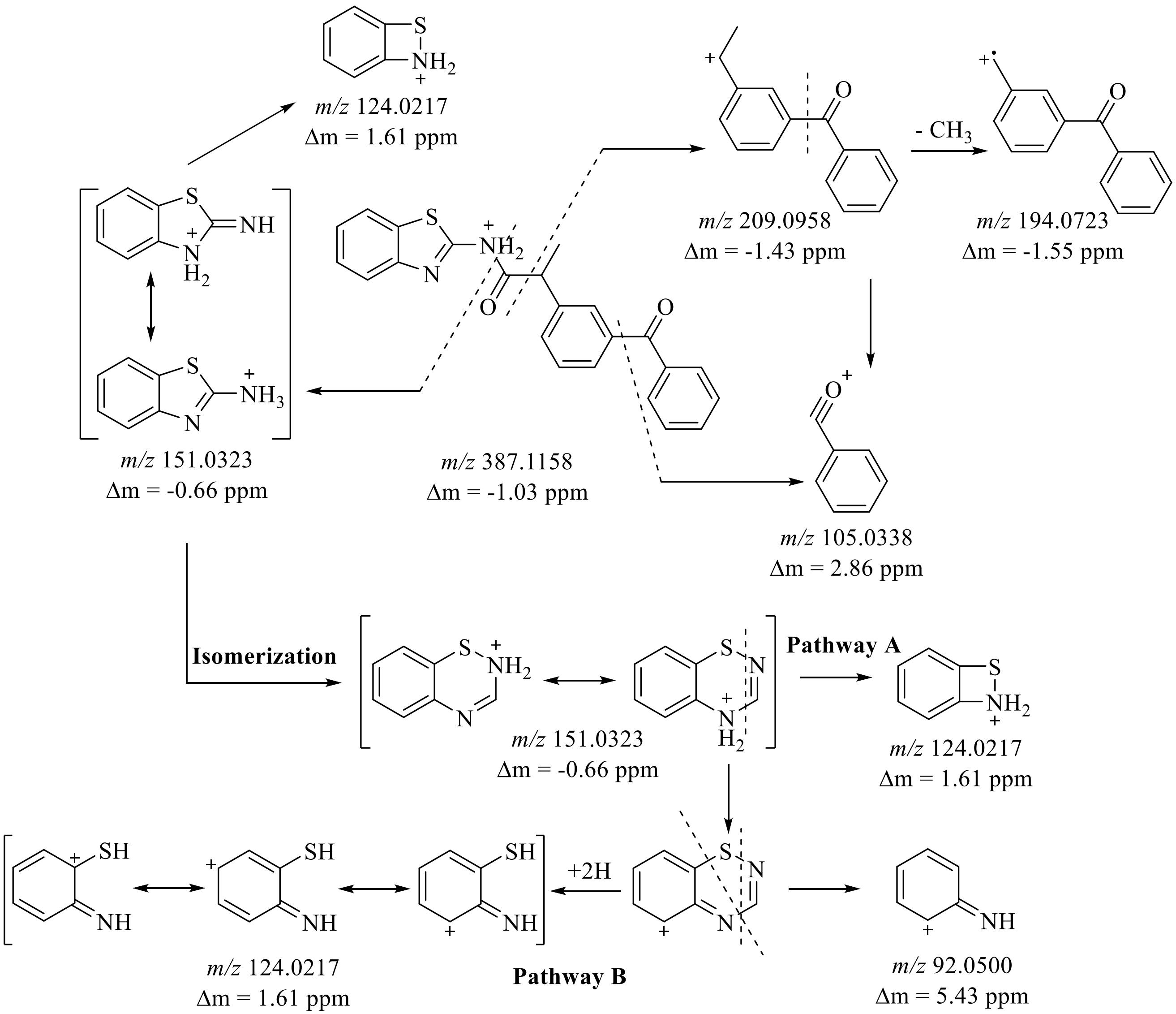

Figure S18. Proposed fragmentation of protonated **3b**.

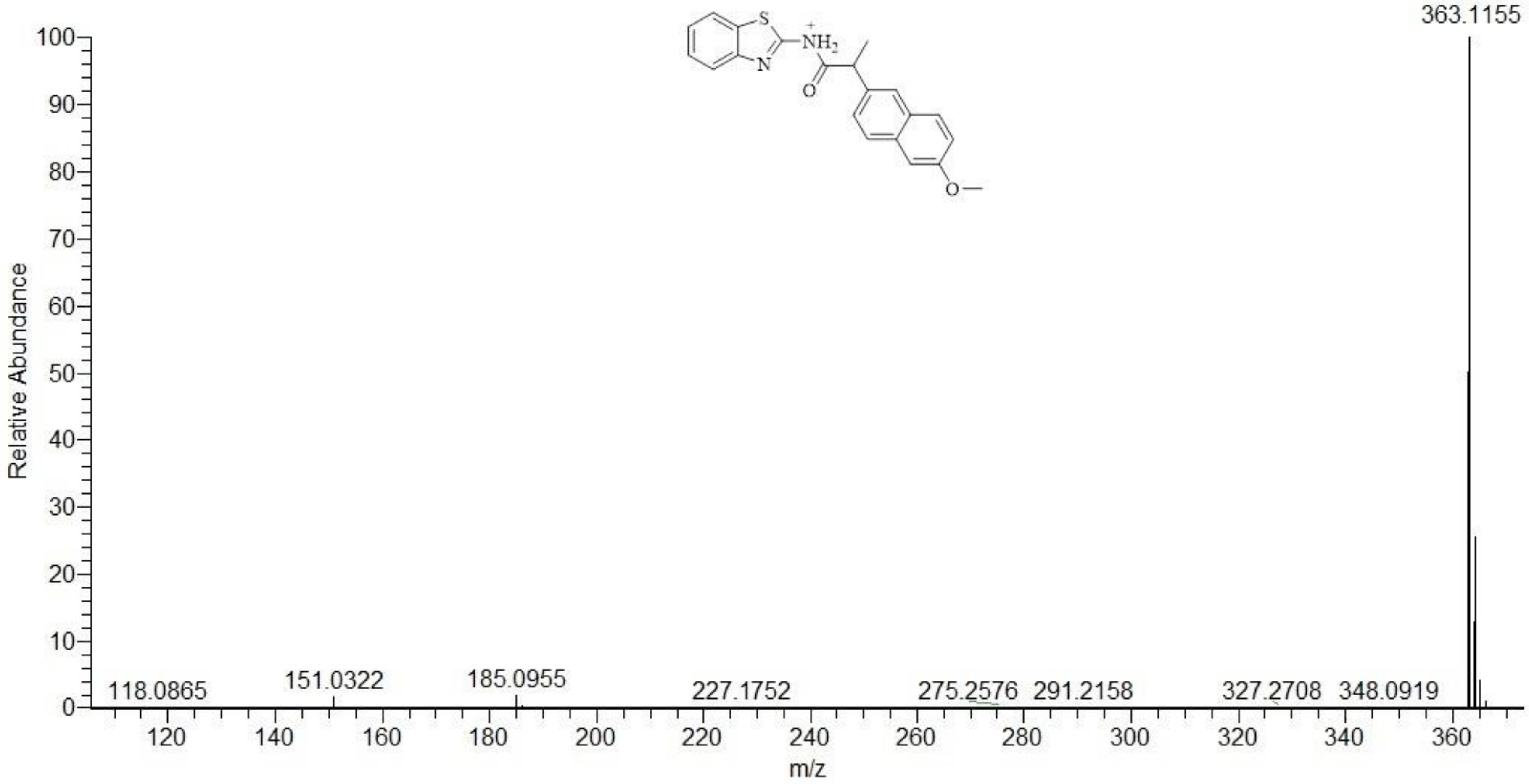

Figure S19. ESI-HRMS of compound **3c**.

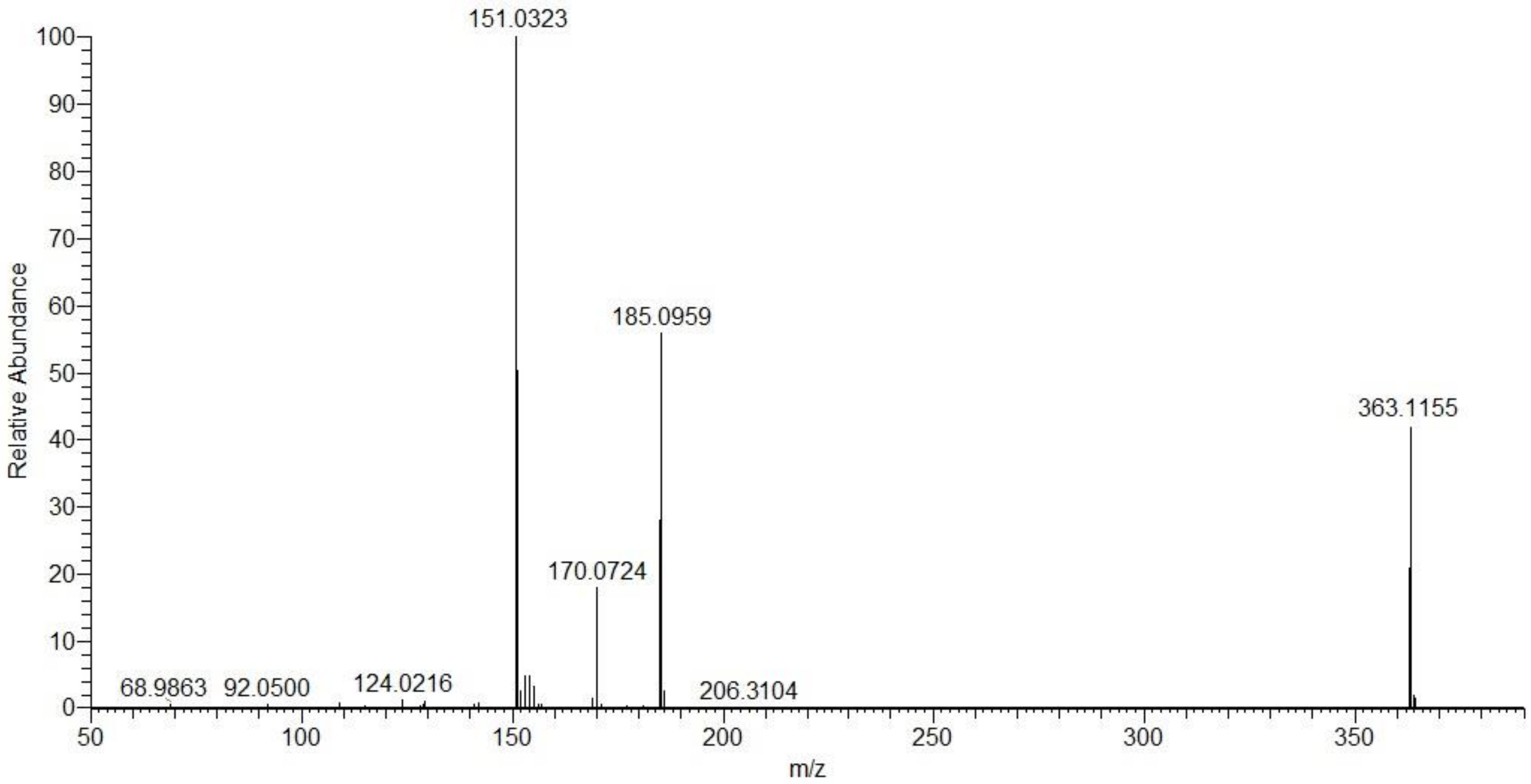

Figure S20. Mass spectrum of **3c** obtained by positive ion ESI-MS/MS.

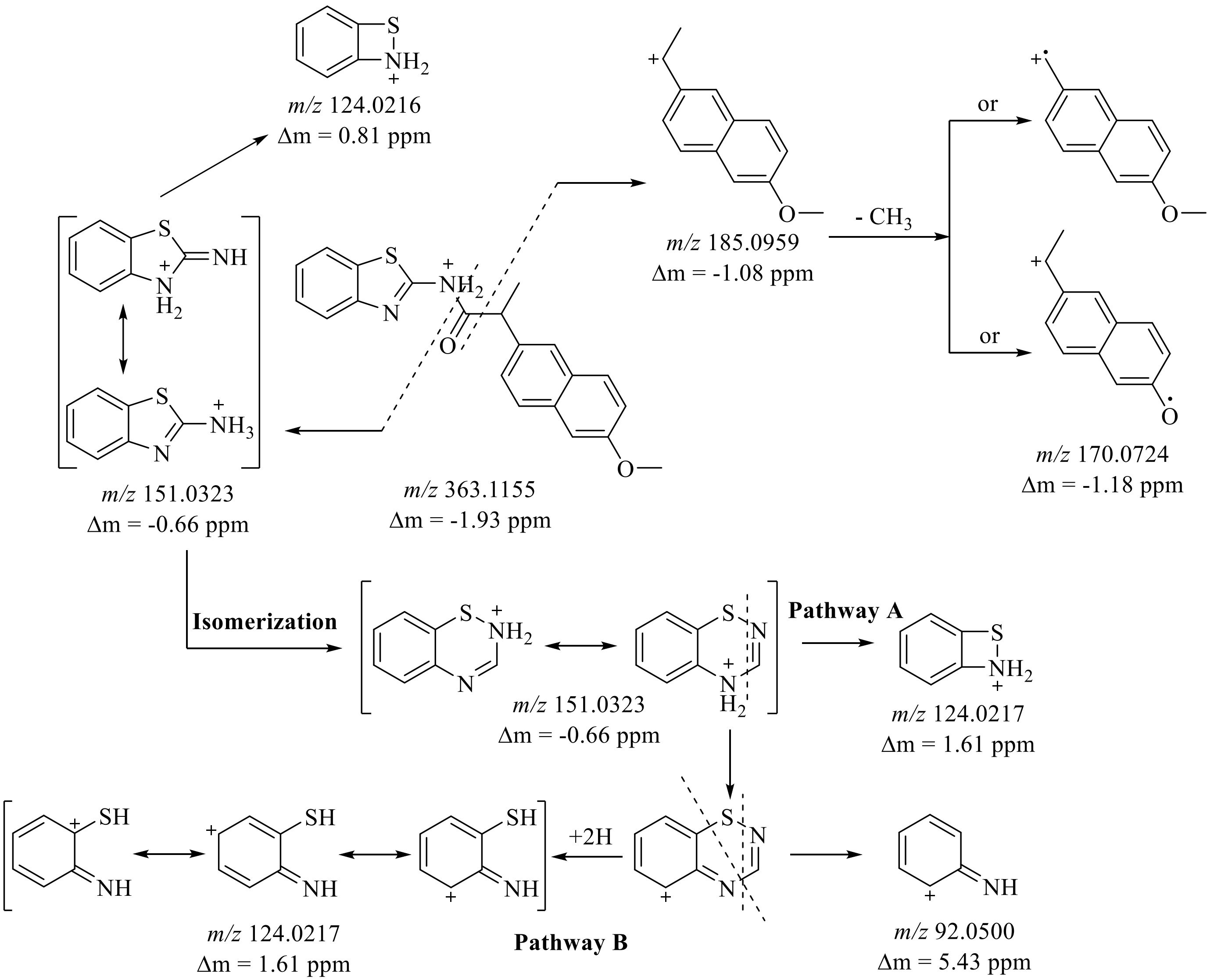

Figure S21. Proposed fragmentation of protonated **3c**.

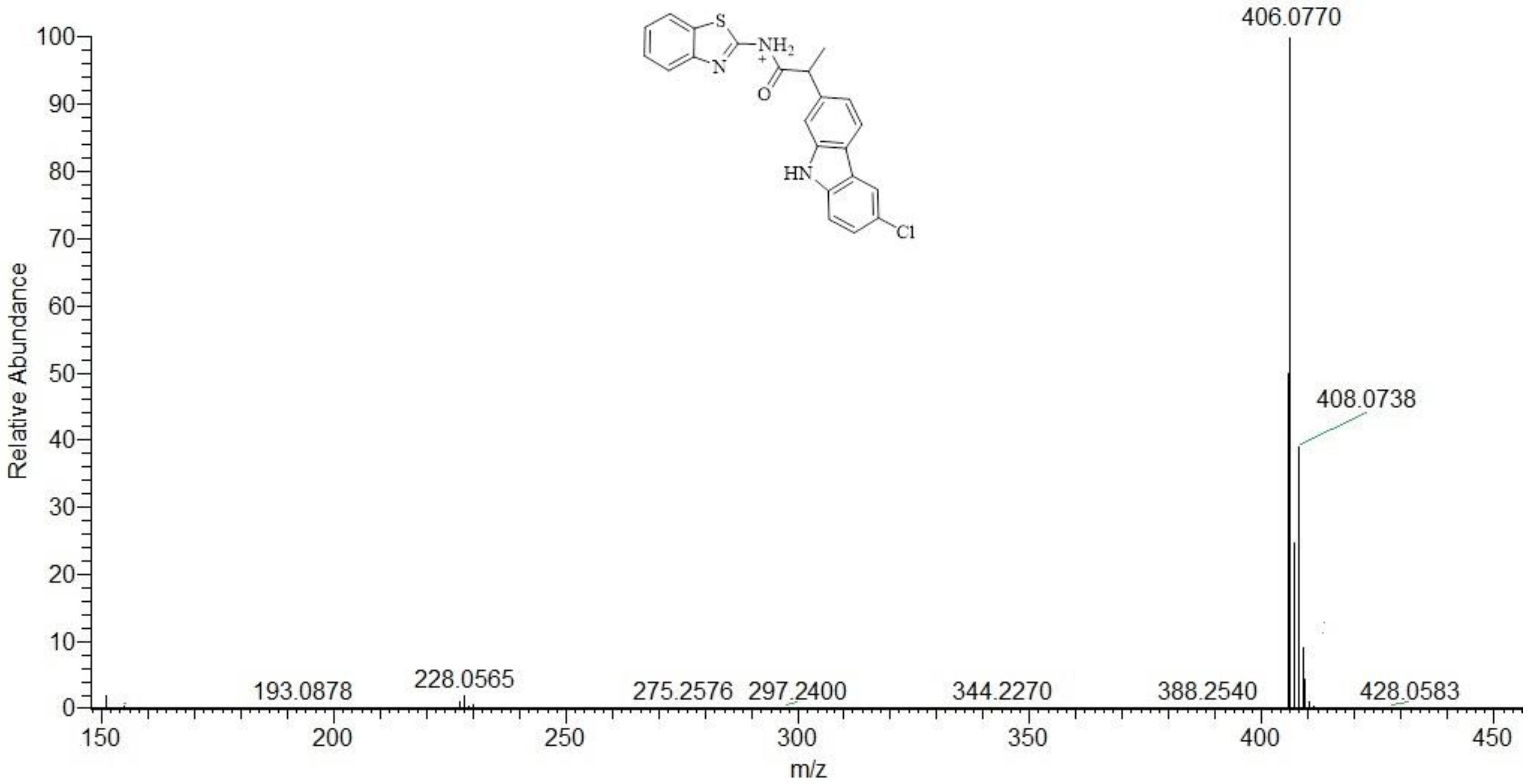

Figure S22. ESI-HRMS of compound **3d**.

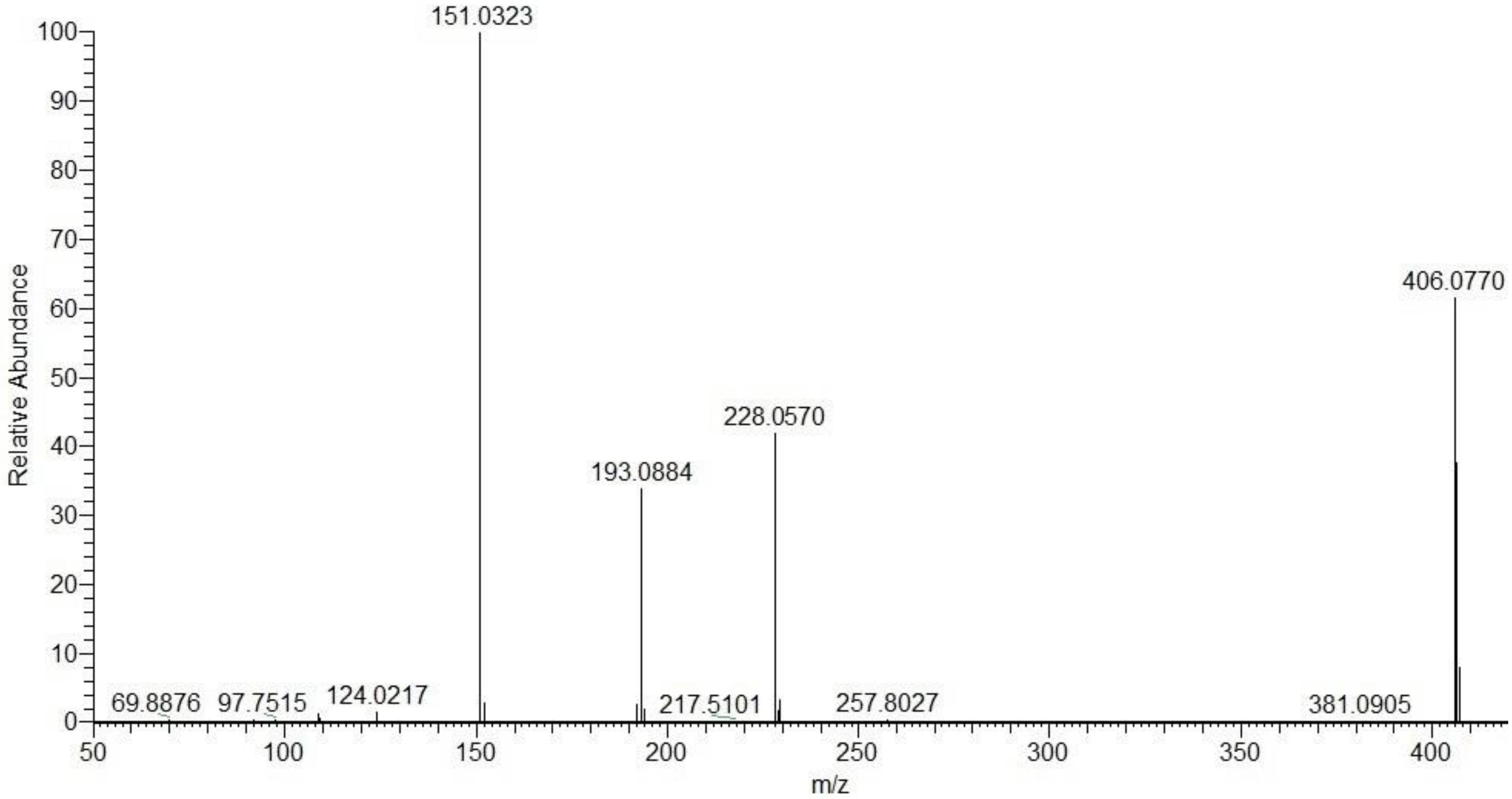

Figure S23. Mass spectrum of **3d** obtained by positive ion ESI-MS/MS.

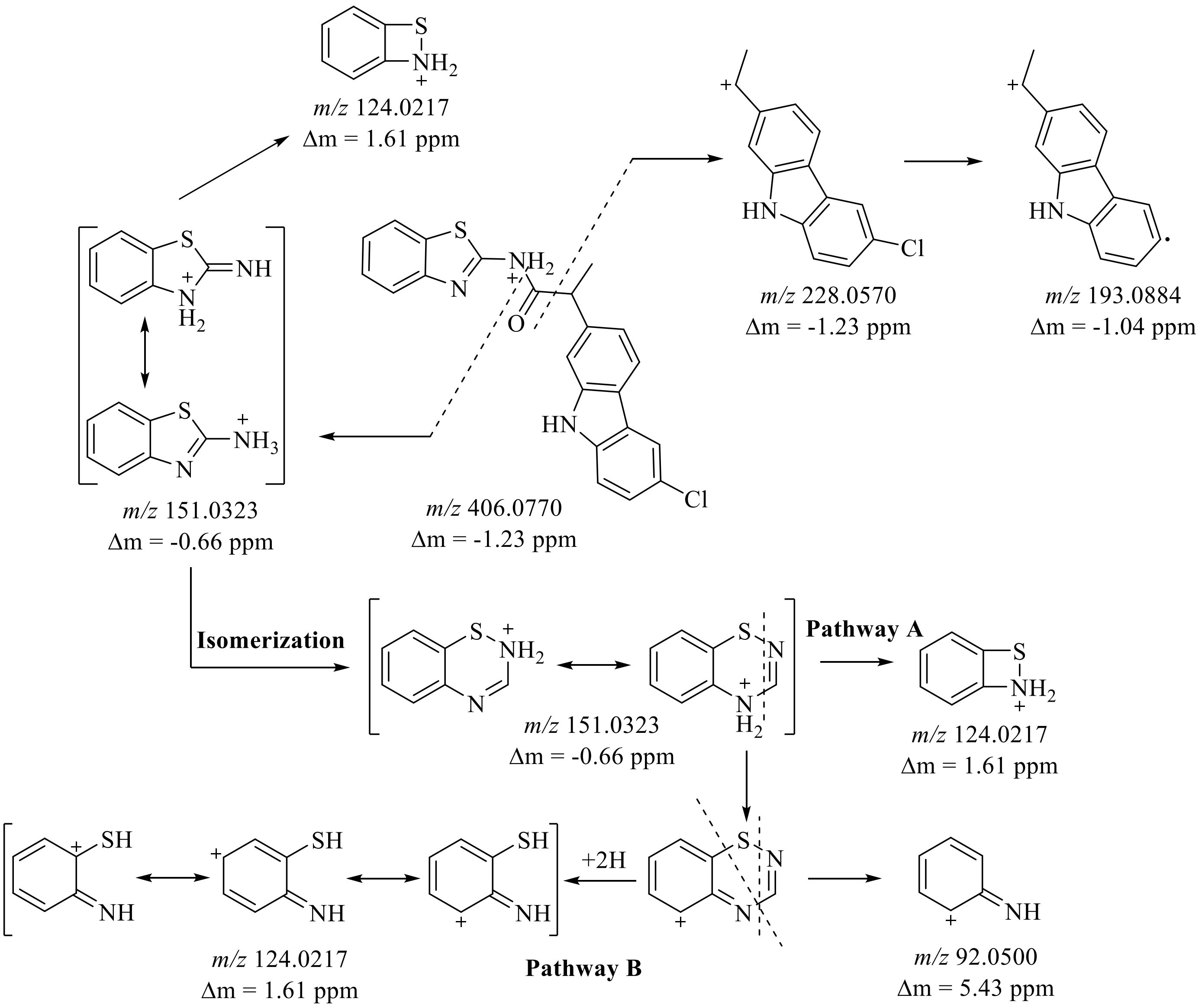

Figure S24. Proposed fragmentation of protonated **3d**.

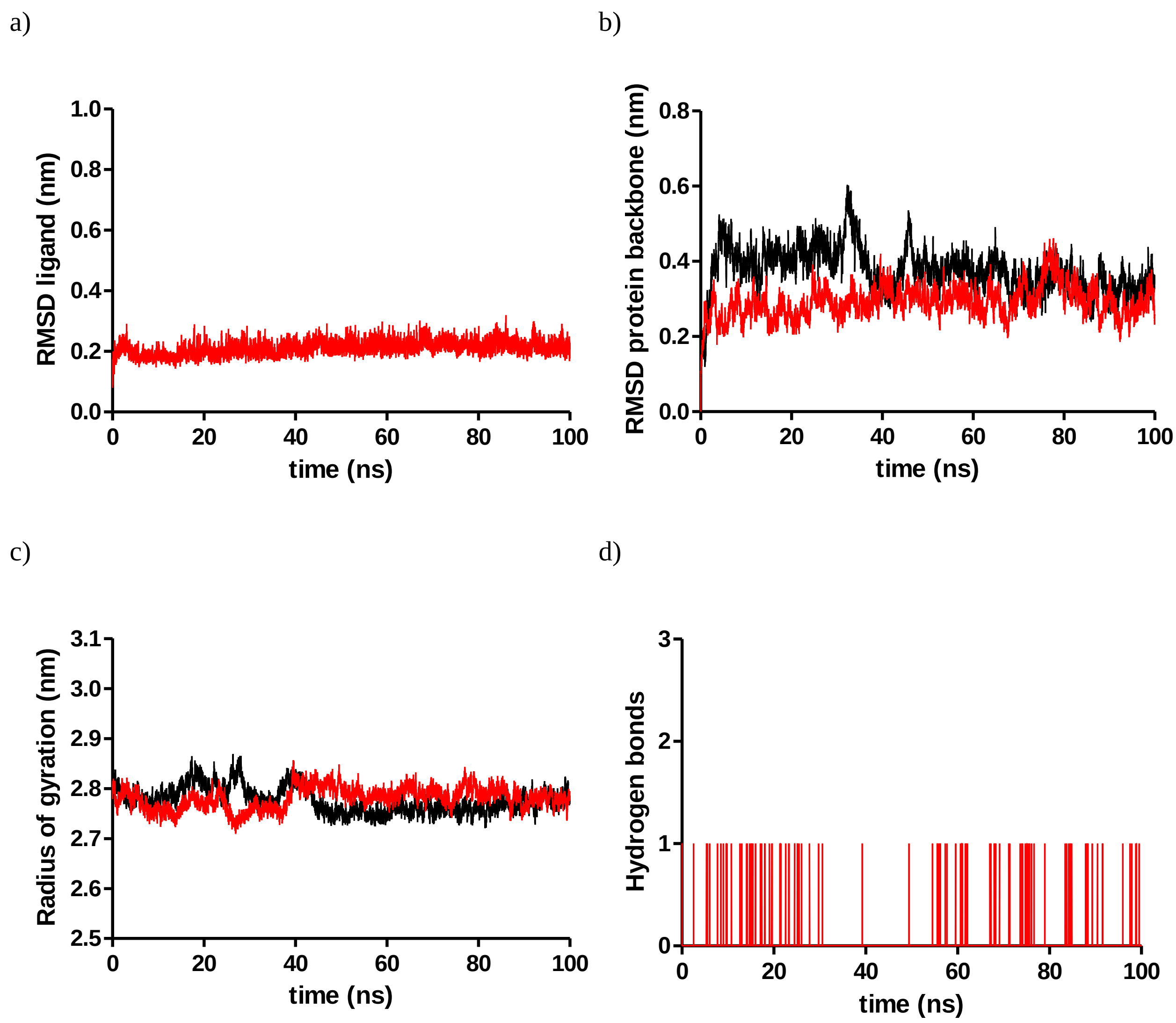

Figure S25. Stability during the molecular dynamics study of the complex of **3b[R]** docked in Sudlow's site I of HSA. a) RMSD of **3b[R]**, b) RMSD of HSA backbone apo (black) and in complex with **3b[R]** (red), c) RG of HSA backbone apo (black) and in complex with **3b[R]** (red), d) hydrogen bonds between **3b[R]** and HSA.

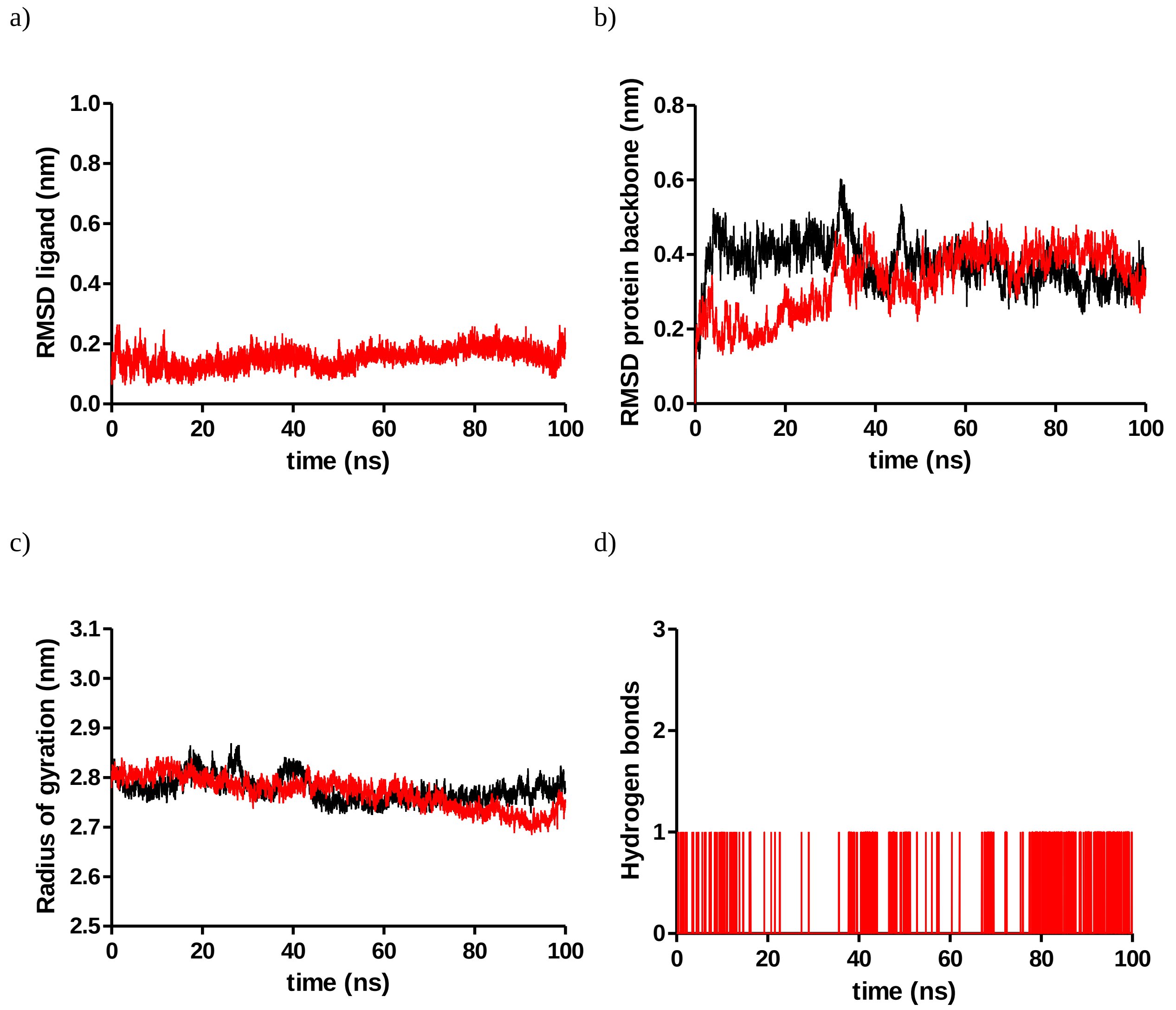

Figure S26. Stability during the molecular dynamics study of the complex of **3e[S]** docked in Sudlow's site II of HSA. a) RMSD of **3e[S]**, b) RMSD of HSA backbone apo (black) and in complex with **3e[S]** (red), c) RG of HSA backbone apo (black) and in complex with **3e[S]** (red), d) hydrogen bonds between **3e[S]** and HSA.

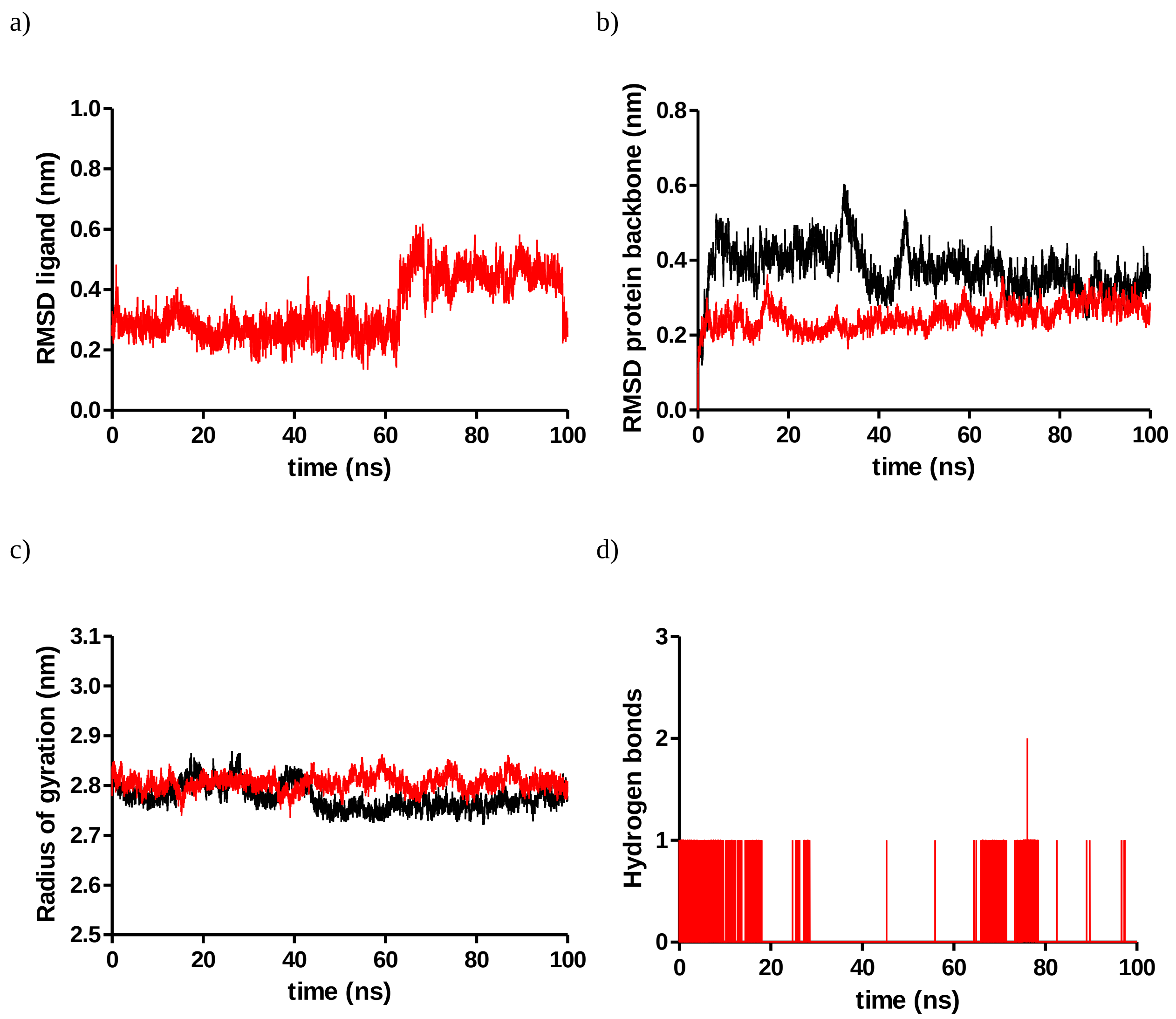

Figure S27. Stability during the molecular dynamics study of the complex of **3b[R]** docked in site III of HSA. a) RMSD of **3b[R]**, b) RMSD of HSA backbone apo (black) and in complex with **3b[R]** (red), c) RG of HSA backbone apo (black) and in complex with **3b[R]** (red), d) hydrogen bonds between **3b[R]** and HSA.

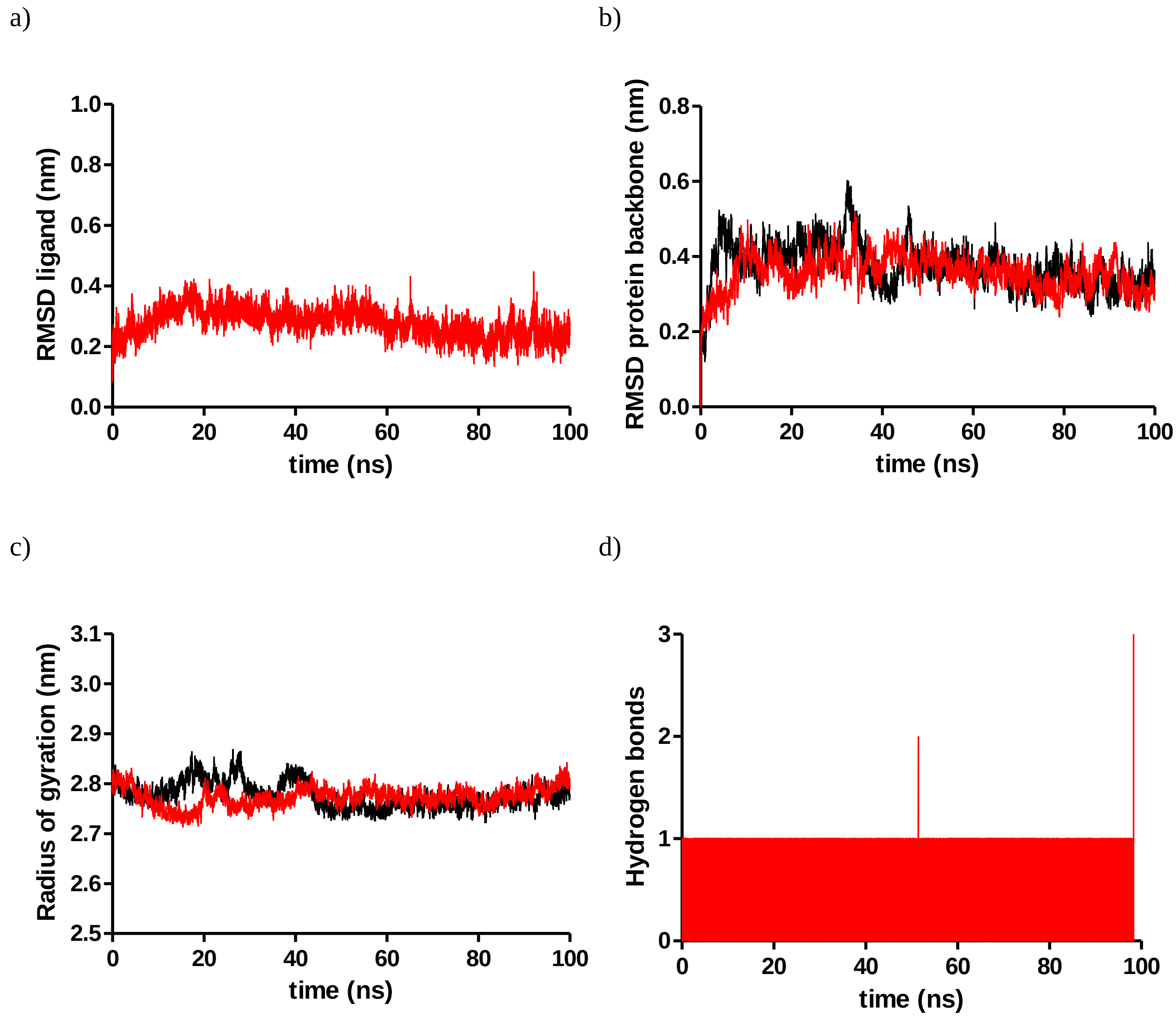

Figure S28. Stability during the molecular dynamics study of the complex of **3e[S]** docked in the cleft site of HSA. a) RMSD of **3e[S]**, b) RMSD of HSA backbone apo (black) and in complex with **3e[S]** (red), c) RG of HSA backbone apo (black) and in complex with **3e[S]** (red), d) hydrogen bonds between **3e[S]** and HSA.

Table S1. The *in vitro* outcomes of the performed biological assays are summarized here. The results for antioxidant activity (HPSA), Hydroxyl radical scavenging activity (HRSA), and inhibition of albumin denaturation (IAD) are expressed in terms of IC_{50} values. Ascorbic acid (AA), quercetin (Qrc), and ibuprofen (Ibu) were used as reference compounds. R_M, denoting lipophilicity, is a dimensionless measure derived from thin-layer chromatography and is dependent on the R_f value.

**Compounds**	**HPSA**	**HRSA**	**IAD**	$R_M \pm SD$	***cLogP***
		$IC_{50} \pm SD$, *µg/mL*			
**3a**	65.51 ± 0.30	375.06 ± 14.62	159.94 ± 2.79	1.629 ± 0.009	5.79
**3b**	60.24 ± 0.47	285.13 ± 27.44	54.64 ± 0.44	1.941 ± 0.010	4.87
**3c**	67.71 ± 0.08	317.91 ± 11.61	64.44 ± 1.61	2.020 ± 0.046	4.93
**3d**	67.22 ± 0.45	551.44 ± 28.48	64.89 ± 1.01	1.755 ± 0.014	6.09
**3e**	63.05 ± 0.28	429.59 ± 21.49	59.24 ± 0.22	1.736 ± 0.015	5.76
**Standards**					
**AA**	24.84 ± 0.35	129.84 ± 4.52	-	-	-
**Qrc**	69.25 ± 1.82	149.18 ± 9.27	-	-	-
**Ibu**	-	-	76.05 ± 1.04	1.110 ± 0.010	3.72
